# Qing-Kai-Ling oral liquid alleviated pneumonia via regulation of intestinal flora and metabolites in rats

**DOI:** 10.3389/fmicb.2023.1194401

**Published:** 2023-06-09

**Authors:** Hongying Chen, Siju Li, Biyan Pan, Kun Liu, Hansheng Yu, Chong Ma, Huiyuan Qi, Yuefeng Zhang, Xinyi Huang, Dongsheng Ouyang, Zhiyong Xie

**Affiliations:** ^1^Department of Clinical Pharmacology, Xiangya Hospital, Central South University, Changsha, China; ^2^School of Pharmaceutical Sciences, Sun Yat-sen University, Shenzhen, China; ^3^Guangzhou Baiyunshan Mingxing Pharmaceutical Company Limited, Guangzhou, China; ^4^Hunan Key Laboratory of Pharmacogenetics, Xiangya Hospital, Institute of Clinical Pharmacology, Central South University, Changsha, China; ^5^Hunan Key Laboratory for Bioanalysis of Complex Matrix Samples, Changsha, China

**Keywords:** Qing-Kai-Ling, pneumonia, intestinal flora, metabolome, transcriptome, multi-omics

## Abstract

**Background:**

Qing-Kai-Ling (QKL) oral liquid, evolving from a classical Chinese formula known as An-Gong-Niu-Huang pills, is a well-established treatment for pneumonia with its mechanism remaining muddled. Studies have shown that the regulation of both intestinal flora and host-microbiota co-metabolism may contribute to preventing and treating pneumonia. The study aimed to investigate the potential mechanism by which QKL alleviates pneumonia from the perspective of ‘microbiota-metabolites-host’ interaction.

**Methods:**

We evaluated the therapeutic effects of QKL on lipopolysaccharide (LPS)-induced pneumonia rats. To explore the protective mechanism of QKL treatment, a multi-omics analysis that included 16S rDNA sequencing for disclosing the key intestinal flora, the fecal metabolome to discover the differential metabolites, and whole transcriptome sequencing of lung tissue to obtain the differentially expressed genes was carried out. Then, a Spearman correlation was employed to investigate the association between the intestinal flora, the fecal metabolome and inflammation-related indices.

**Results:**

The study demonstrated that pneumonia symptoms were significantly attenuated in QKL-treated rats, including decreased TNF-α, NO levels and increased SOD level. Furthermore, QKL was effective in alleviating pneumonia and provided protection equivalent to that of the positive drug dexamethasone. Compared with the Model group, QKL treatment significantly increased the richness and αlpha diversity of intestinal flora, and restored multiple intestinal genera (e.g., *Bifidobacterium*, *Ruminococcus_torques_group*, *Dorea*, *Mucispirillum*, and *Staphylococcus*) that were correlated with inflammation-related indices. Interestingly, the intestinal flora demonstrated a strong correlation with several metabolites impacted by QKL. Furthermore, metabolome and transcriptome analyses showed that enrichment of several host-microbiota co-metabolites [arachidonic acid, 8,11,14-eicosatrienoic acid, LysoPC (20:0/0:0), LysoPA (18:0e/0:0), cholic acid, 7-ketodeoxycholic acid and 12-ketodeoxycholic acid] levels and varying lung gene (*Pla2g2a*, *Pla2g5*, *Alox12e*, *Cyp4a8*, *Ccl19*, and *Ccl21*) expression were observed in the QKL group. Moreover, these metabolites and genes were involved in arachidonic acid metabolism and inflammation-related pathways.

**Conclusion:**

Our findings indicated that QKL could potentially modulate intestinal flora dysbiosis, improve host-microbiota co-metabolism dysregulation and regulate gene expression in the lungs, thereby mitigating LPS-induced pneumonia in rats. The study may provide new ideas for the clinical application and further development of QKL.

## 1. Introduction

Pneumonia, belonging to a respiratory infection disease, is the fourth major cause of death globally (Zhang et al., 2022). Increasing evidence has demonstrated that pneumonia is caused by multiple factors, among which intestinal flora dysbiosis is recognized as a vital cause (Li et al., 2020; Deng et al., 2021). Intestinal flora dysbiosis occurs in pneumonia patients as well as animal models (Feng et al., 2020; Zuo et al., 2020). Furthermore, regulation of intestinal flora and thus therapy in pneumonia can be achieved by the administration of probiotics (e.g., *Bacillus subtilis* and *Enterococcus faecalis*) and some Chinese medicines (CMs), such as Qing-Fei-Pai-Du decoction and Xuan-Bai-Cheng-Qi decoction (Manzanares et al., 2016; Zeng et al., 2016; Huo et al., 2022; Wu et al., 2022), which further suggests that regulation of intestinal flora disorders may be a promising approach for the prevention and treatment of pneumonia. Additionally, due to the interaction between intestinal microbiota and lung, intestinal flora dysbiosis can exacerbate lung inflammation (Dang and Marsland, 2019; Zhang D. et al., 2020). More specifically, intestinal flora dysbiosis raises the possibility of a weakened intestinal barrier, which in turn increases the risk of microbial translocation to the lungs. Meanwhile, pro-inflammatory mediators enter the bloodstream and worsen lung inflammation (Tang et al., 2021; Synodinou et al., 2022). Collectively, prevention and treatment of pneumonia depend not only on the modulation of gut microbiota dysbiosis but also on the regulation of the interaction between intestinal flora and lung. On the other hand, intestinal flora can communicate with the host through various host-microbiota co-metabolites, such as short-chain fatty acids and bile acids (Winston and Theriot, 2020; Zhou et al., 2021), and thus co-regulate lung diseases (Ma et al., 2022). In addition, metabolic abnormalities have also been implicated in the pathogenesis of pneumonia. There are intestinal flora disturbances and dysregulation of host metabolism in pneumonia animals (Wu et al., 2022), and CMs exert therapeutic effects on pneumonia through potential mechanisms associated with the regulation of ‘microbiota-metabolites-host’ interaction (Lian et al., 2022). Thus, targeting intestinal flora disorders and metabolic abnormalities could be developed as a novel strategy for preventing and treating pneumonia.

Qing-Kai-Ling (QKL) oral liquid, consisting of eight drugs, including *Margaritifera Concha*, *Gardenia Jasminoide*, *Buffalo Horn*, *Radix isatidis*, *Baicalin*, *Flos Lonicerae*, *cholic acid*, and *hyodeoxycholic acid* (Zhang et al., 2006), is a widely used drug to treat pneumonia in China. Compared with traditional drugs, including antibiotics (e.g., fluoroquinolones, cephalosporins and macrolides) and vaccines (Torres et al., 2021), QKL effectively sheers away corresponding side effects, such as drug resistance, complicated syndromes, high costs and inadequate coverage. In recent years, some researches have investigated the mechanisms by which QKL protects against pneumonia. For instance, QKL has been shown to inhibit TLR4/NF-κB pathway activation, thereby preventing and treating pneumonia (Chen et al., 2022). Furthermore, a network pharmacology research suggests that QKL treats COVID-19 with anti-inflammation, cytokine storm remission, improvement in immunity, etc. (Zhang Y. R. et al., 2020). Moreover, polyphenolic compounds (chlorogenic acid and caffeic acid), flavonoids (baicalin) and other components (indigo and indirubin), the main bioactive components of QKL, can alleviate multiple types of inflammation by modulating intestinal flora (Zhang et al., 2017; Peng et al., 2021; Wan et al., 2021; Yang et al., 2021). Interestingly, the underlying mechanism by which QKL treats pneumonia is still not fully understood, and whether QKL ameliorates pneumonia through modulating intestinal flora, and further regulating host-microbiota co-metabolites has not been elucidated.

Herein, we evaluated the effects of QKL treatment in a pneumonia rat model induced by lipopolysaccharide (LPS) and investigated the potential mechanism of QKL in treating pneumonia at a comprehensive level using multi-omics analyses. Specifically, the technologies including 16S rDNA sequencing, UPLC-Q-TOF-MS/MS technology and RNA sequencing were employed to reveal how QKL affected the intestinal flora, co-metabolites produced by host-microbiota, and lung gene expression, respectively. Moreover, a Spearman correlation was utilized to investigate the association between fecal microbiota and metabolome data. The study aims to shed light on the clinical application of QKL as an anti-pneumonia drug, offering fresh ideas for its potential use.

## 2. Materials and methods

### 2.1. Preparation of drugs

Qing-Kai-Ling oral liquid was supplied by Guangzhou Baiyunshan Mingxing Pharmaceutical Co., Ltd. (Guangzhou, China). It was extracted as a mixture including *Margaritifera Concha* (50 g), *Gardenia Jasminoide* (25 g), *Buffalo Horn* (25 g), *Radix Isatidis* (200 g), *Baicalin* (5 g), *Flos Lonicerae* (60 g), *cholic acid* (3.25 g), and *hyodeoxycholic acid* (3.75 g) in 1,000 mL of sterile water. The above 1,000 mL QKL oral liquid was further concentrated into 500 mL liquids for animal experiments.

### 2.2. Animal experiments

**FIGURE 1:**
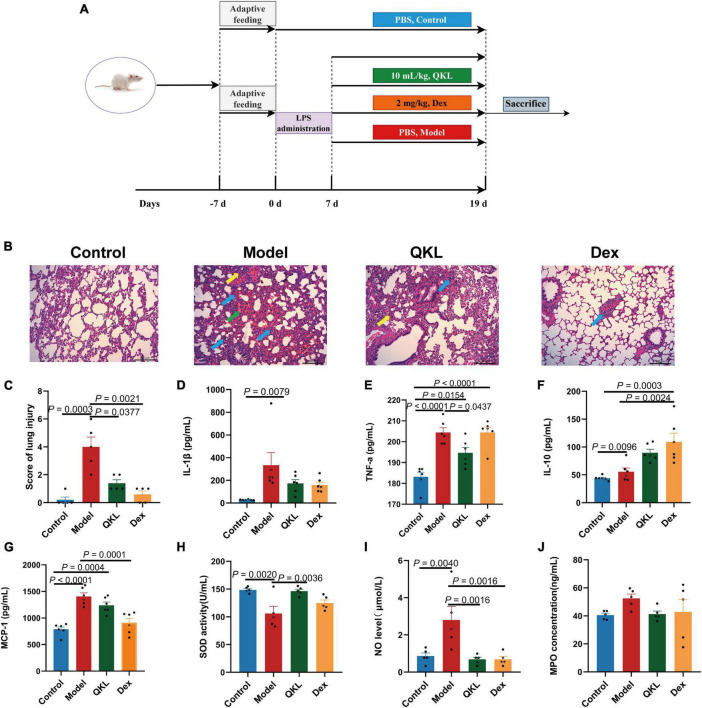
Effects of QKL treatment on LPS-induced pneumonia rats. **(A)** The animal experiment flowchart. **(B)** Representative H&E images of lung obtained from groups. Green arrows represent hemorrhage, yellow arrows represent thickened alveolar septum, and blue arrows represent infiltration of the lung interstitium (scale bar: 200 μm). **(C)** H&E score of lung injury (*n* = 5 each group). **(D–G)** The contents of IL-1β, TNF-α, IL-10, and MCP-1 in plasma using a Bio-Plex 200 chip system (*n* = 6 per group). **(H–J)** The levels of SOD, NO, and MPO in serum (*n* = 5 per group).

Male Sprague-Dawley (SD) rats weighing 170–200 g were supplied by Bes Test Bio-Tech Co., Ltd. (Zhuhai, China). The scheme was authorized by the Laboratory Animal Center of Sun Yat-sen University (ethics No: SYSU-IACUC-2021-000511). After a 7-day acclimation period, 24 SD rats were randomly allocated to the Control group, Model group, QKL group and dexamethasone (Dex) group (*n* = 6 in each group). Lipopolysaccharide (LPS) was continuously injected intraperitoneally into rats (4 mg/kg/day) for 7 days to induce pneumonia except in the Control group. After model establishment for 7 days, the rats in the QKL and Dex groups were orally treated with QKL (10 mL/kg/day) and Dex (2 mg/kg/day), respectively, for 12 days. While the other two groups received equal volumes of sterile PBS buffer by gavage for 12 days. On the 19th days, rat feces were collected using metabolic cages with ice-immersed 10 mL EP tubes underneath. Rats were sacrificed under anesthesia with ether after overnight fasting. Blood samples (plasma and serum), lung tissue and intestinal contents of rats were collected and preserved at −80°C. The whole animal experiment process was depicted in Figure 1A.

### 2.3. Pathological observation of lung tissue

The right upper lung tissue was excised, immersed in 10% formaldehyde, and embedded in paraffin. Then, sliced 5-μm sections were stained using hematoxylin and eosin (H&E). A semi-quantitative score was employed to assess lung tissue lesions, including edema, inflammation of alveolar and interstitial tissues, and hemorrhage of alveolar and interstitial tissues. The grades were as follows: no injury, mild injury, mild to moderate injury, moderate to severe injury and severe injury, representing grades 0, 1, 2, 3, and 4, respectively (Rotta et al., 1999).

### 2.4. Detection of biochemical indices in blood samples

The levels of four inflammatory cytokines in plasma, including IL-1β, TNF-α, IL-10, and monocyte chemotactic protein-1 (MCP-1), were measured using the Bio-Plex 200 chip system (Bio-Rad Laboratories, CA, United States). Additionally, two oxidative stress indices in serum, superoxide dismutase (SOD) and nitric oxide (NO), were detected by testing kits (Nanjing Jiancheng Biotechnology, Nanjing, China). The content of myeloperoxidase (MPO) in serum was detected by an enzyme-linked immunosorbent assay kit (Cloud-Clone Corp., Houston, TX, United States). All procedures were operated in accordance with the manuals of the manufacturer.

### 2.5. 16S rDNA gene sequencing

The microbiome of 18 feces samples (the Control, Model and QKL groups with 6 samples in each group) was for testing. Bacterial DNA was extracted from the samples using the HiPure Stool DNA Kits from Magen, China. Then, the V3-V4 regions of bacterial gene were amplified by the primer pairs: 341F (5′-CCTACGGGNGGCWGCAG-3′) and 806R (5′-GGACTACHVGGGTATCTAAT-3′). After the amplified DNA was purified and detected, the obtained amplicons were mixed in equimolar amounts and sequenced using the Illumina 2500 platform at Gene *Denovo* Bio-Tech Co., Ltd., in Guangzhou, China. Then, the sequenced data treatment was as follows: raw reads obtained from the sequencing were filtered using FASTP v0.18.0 to acquire clean reads. Then paired and clean reads were merged as raw tags using FLASH v1.2.11, and noisy sequences of raw tags were also further filtered. Furthermore, the obtained clean tags were classified into operational taxonomic units (OTUs) of more than 97% similarity. The obtained OTU sequences were annotated using the RDP Classifier. Finally, a PICRUSt analysis was conducted to infer the ecological functions of intestinal microbial communities by predicting gene functions. As a result, the sequencing data yielded between 22,038 and 50,820 sequences per sample, with a minimum length of 201 bp and a maximum length of 469 bp. These data could be employed to analyze the composition and diversity of the intestinal flora and to identify alterations that may be associated with QKL treatments.

### 2.6. Metabolic analysis

#### 2.6.1. Sampling and processing

A one mL acetonitrile (ACN)/H_2_O (4:1, v/v) mixture was added to fecal samples (100 ± 2 mg) collected on day 19 in a 2 mL EP tube. The combination was vortexed for 5 min. Then, after 3 times freeze-thaw and 10 cycles of ultrasonication were conducted, the samples were centrifuged at 4,500 rpm at 4°C for 10 min to obtain the upper layer. Finally, the supernatant of the fecal extract was determined using ultra-high performance liquid chromatography with quadrupole time of flight mass spectrometry/mass spectrometry (UPLC-Q-TOF-MS/MS) analysis. Additionally, we also obtained a quality control (QC) sample by blending 18 pieces with an equivalent volume (20 μL).

#### 2.6.2. UPLC-Q-TOF-MS/MS analysis

In order to separate the fecal samples, we employed an ACQUITY HSS T3 column (100 mm × 2.1 mm, 1.8 μm) with a flow rate of 0.4 mL/min and an injection volume of 1 μL. In addition, the column temperature was 40°C. The mobile phases included A (water with 0.1% formic acid) and B (acetonitrile with 0.1% formic acid). The gradient elution was: (1) 0 min, 1% B; (2) 1 min, 1% B; (3) 3.5 min, 15% B; (4) 7.5 min, 25% B; (5) 9 min, 35% B; (6) 11.5 min, 99.9% B; (7) 17 min, 99.9% B; and (8) 20 min, 1% B. The ion source of mass was electron spray ionization (ESI). Moreover, MS was run under ESI+ and ESI– modes, with capillary voltages of 2.5 KV and 2 KV, respectively. The MS data were collected in the range of 50–1,000 m/z with a trap collision energy from 20 eV to 50 eV. Other parameters were set as follows: cone voltage: 40 V; drying gas (nitrogen) and ion source temperatures were 350°C and 120°C, respectively.

#### 2.6.3. Metabolome data analysis

The raw data were collected using MassLynx v4.1 before they were imported into Progenesis QI v2.0, which completed peak alignment, normalization, retention time correction, and compound identification. The processed data were then analyzed by multivariate statistical analysis (PLS-DA and OPLS-DA) in SIMCA v14.1. R^2^Y and *Q*^2^ are generally utilized to evaluate the interpretation rate and the predictive ability of models, respectively. To assess the relative importance of variables in the OPLS-DA model, variable importance in the projection (VIP) is a strategy with a threshold of 1. ANOVA test along with a threshold of *P* < 0.05 was employed to evaluate the significance of peak area among groups. The Benjamini-Hochberg FDR method was then used to adjust the *P-*values. Taken together, compounds with cut-off values (VIP > 1.0 and *P* < 0.05) were more likely to be regarded as differential metabolites. The metabolites that coincided with the mentioned criteria were subject to further identification using both the HMDB online website^1^ and the KEGG database^2^, and the mass error between theoretical and measured values of screened compounds was less than ± 5 ppm. Furthermore, metabolites that demonstrated the abovementioned characteristics and were presented in both the comparison of the Control and Model groups, as well as the Model and QKL groups, were considered potential biomarkers. The KEGG pathway analysis was obtained by MetaboAnalyst v5.0.^3^

#### 2.6.4. Transcriptome analysis of lung tissue

The lung tissue from the Control, Model and QKL groups was picked for transcriptome sequencing. Total RNA extraction was performed with the TRIzol reagent kit (Invitrogen, USA). The integrity and quality of nine RNA samples were examined utilizing agarose gel electrophoresis and Agilent 2100 bioanalyzers (Agilent Technologies, USA). Sequencing libraries were then constructed and the RNA sequencing was completed by Gene *Denovo* Bio-Tech Co., Ltd. Subsequently, based on the sequenced and processed data, differential analysis, protein-protein network (PPI), and KEGG enrichment analysis of differentially expressed genes (DEGs) were executed based on the sequenced and processed data.

### 2.7. Statistical analysis

Data were presented as the mean ± SEM. Using OriginPro v9.1.0 and GraphPad Prism v9.0 to analyze and visualize the data. Statistical differences among groups were analyzed using one-way analysis of variance (ANOVA) followed by Tukey’s test, and non-normal distribution data were analyzed by non-parametric test.

## 3. Results

### 3.1. Characterization of components in QKL

In order to investigate the components of QKL, ultra performance liquid chromatography-quadrupole time-of-flight-mass spectrometry (UPLC-Q-TOF-MS) was employed. And total ion chromatography (TIC) of QKL in ESI+ and ESI– modes was depicted in Supplementary Figure 1. Additionally, totally 46 compounds of QKL identified were shown in Supplementary Table 1, which included 8 bile acids, 5 flavonoids, 10 iridoids, 9 organic acids, 9 amino acids, and 5 other components.

### 3.2. QKL attenuated symptoms of LPS-induced pneumonia in rats

According to the results of histopathological examination of the lung, we found that the Control group displayed a normal structure of lung tissue. LPS exposure in rats dramatically exacerbated lung lesions, including: infiltration of the lung interstitium by a rising number of inflammatory cells, widening of the distance between the alveolar septum, alveolar disarray and alveolar hemorrhage (Figure 1B). However, the QKL group showed significant improvement of lung damage, which caused a significant decline in the histopathologic score (Figure 1C).

The level of inflammatory cytokines is generally upregulated during the development of pneumonia. Compared with the Control group, IL-1β (*P* = 0.0079), TNF-α (*P* < 0.0001), and MCP-1 (*P* < 0.0001) were significantly higher in the Model group. Additionally, compared with the Model group, QKL-treated rats exhibited a marked decrease in TNF-α (*P* = 0.0437) and a declining tendency in IL-1β, and MCP-1 but without significance (*P* > 0.05) (Figures 1D–G). Simultaneously, the anti-inflammatory cytokine IL-10 was increased in the QKL and Dex groups. Taken together, these findings revealed that QKL acted as an anti-inflammatory agent, effectively suppressing the levels of inflammatory cytokines. LPS-stimulated pneumonia may increase oxidative stress in rats. Therefore, we examined the contents of NO, SOD and MPO. Compared with the Control group, the NO level (*P* = 0.0040) and MPO concentration were increased, while SOD (*P* = 0.0020) was significantly depleted in the Model group. However, QKL treatment not only significantly increased SOD activity (*P* = 0.0036), but also reduced the generation of NO (*P* = 0.0016) and MPO (*P* > 0.05) (Figures 1H–J). In summary, QKL treatment effectively attenuated LPS-induced pneumonia in rats.

### 3.3. QKL caused intestinal flora alterations in rats with pneumonia

**FIGURE 2:**
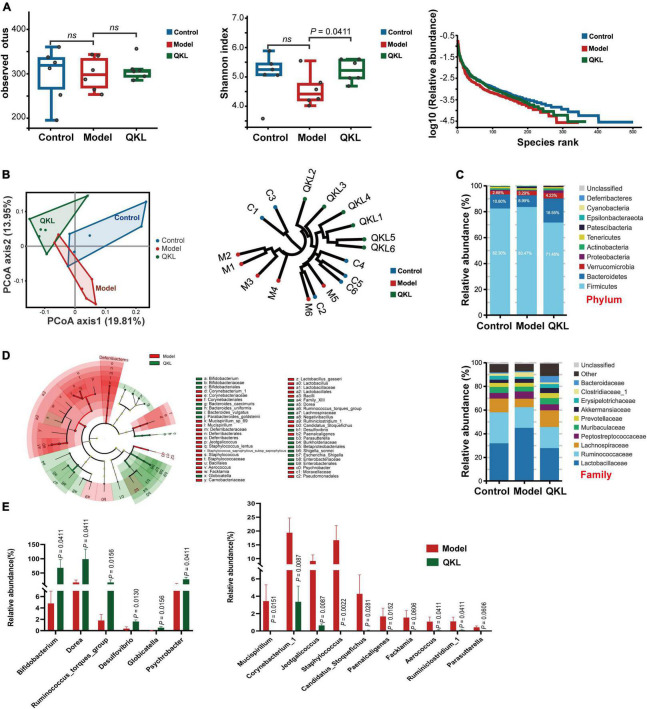
Analysis of fecal microbiota diversity and structural comparison (*n* = 6 per group). **(A)** Observed OTUs, Shannon index and rank abundance curve of bacterial OTUs. **(B)** Principal coordinates analysis of unweighted UniFrac distance matrices, hierarchical clustering based on unweighted UniFrac similarity. **(C)** Bacterial taxonomic profiling in the phylum and family levels of intestinal flora. **(D)** Taxonomic differences of fecal microflora between the Model and QKL groups. **(E)** Boxplots of the relative abundance of 6 up-regulated and 10 down-regulated intestinal flora constituents at the genus level after QKL administration. ns, no significance.

After 16S rDNA sequencing, a total of 1,105,686 raw reads were obtained. Totally 661,452 effective sequences were obtained after removing whole chimeric tags by USEARCH, which remained for further analysis. No apparent changes were observed in bacterial richness among the Control, Model and QKL groups (Figure 2A). However, the Shannon index, reflecting both richness and evenness of intestinal flora, was the highest in the QKL group. Meanwhile, as depicted in the rank abundance curve, the intestinal flora αlpha diversity in the QKL group was between that of the other two groups. Moreover, according to the unweighted UniFrac distance of principal coordinates analysis (PCoA) analysis and hierarchical clustering results, excellent isolation was observed among the three groups, representing an overall structural difference of intestinal flora in rats (Figure 2B). In summary, by contrast with the Model group, the αlpha and beta diversity of the QKL group were significantly altered. Then, we investigated the taxonomic profile of intestinal flora. Two predominant phyla, consisting of Bacteroidetes and Firmicutes, occupied higher than 90% of the cumulative relative abundance (Figure 2C). An increased relative abundance of Bacteroidetes but a lower proportion of Firmicutes, and a smaller ratio of Firmicutes/Bacteroidetes (F/B) (Supplementary Figure 2) were observed in the QKL group as compared to the Model group. Meanwhile, Bacteroidetes, Verrucomicrobia and Proteobacteria were accumulated, while Firmicutes, Cyanobacteria and Deferribacteres were depleted after QKL intervention. At the family level, the differences were assessed by the Wilcoxon test. Compared with the Model group, QKL treatment markedly upregulated the relative abundance of Lachnospiraceae, Bifidobacteriaceae and Akkermansiaceae. In contrast, it significantly reduced the abundance of Lactobacillaceae, Corynebacteriaceae, Staphylococcaceae, Deferribacteraceae and Moraxellaceae (Figure 2C). The linear discriminant analysis effect size (LEfSe) was conducive to further identifying abundance variations of the specific intestinal microbiota and screening the potential microbial biomarkers (Figure 2D). In addition, compared with the Model group, the relative abundance of 16 genera was significantly altered following treatment with QKL, including 6 increasing genera (*Bifidobacterium*, *Dorea*, *Ruminococcus_torques_group*, *Globicatella*, etc.) and 10 decreasing genera (*Mucispirillum*, *Corynebacterium_1*, *Staphylococcus*, *Facklamia*, etc.). Moreover, the 16 genera were identified as the key characteristics (Figure 2E). Namely, the 16 genera were conducive to distinguishing the microbial communities between the Model and QKL groups. Collectively, QKL treatment induced overwhelming changes in community structures of intestinal flora in rats with pneumonia, which might affect metabolism and host health.

**FIGURE 3:**
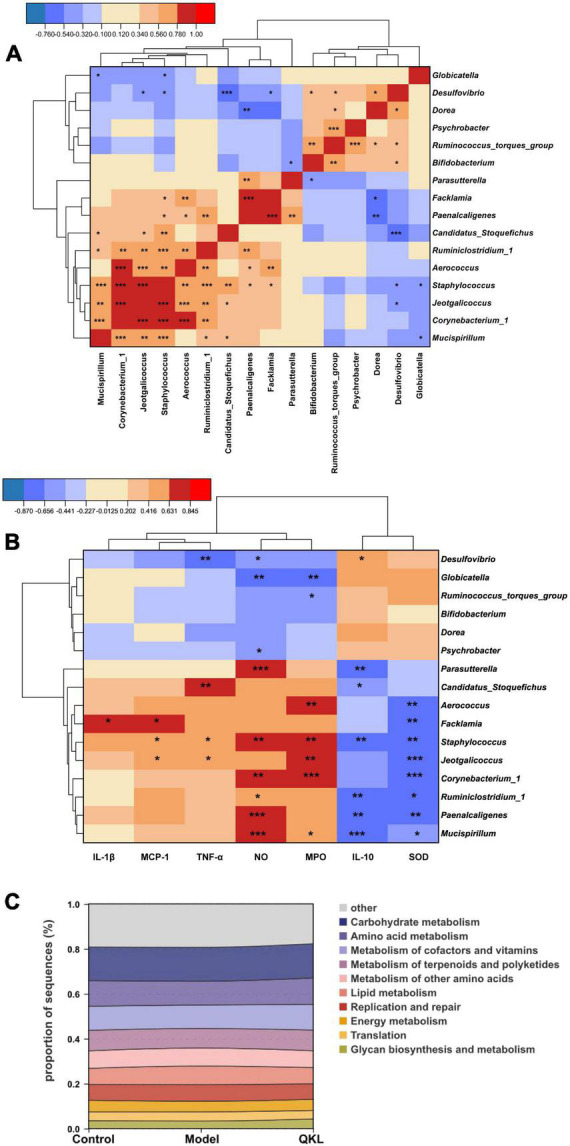
Significantly altered intestinal flora in rats with pneumonia. **(A)** Spearman correlation analysis of 16 discriminant taxa. **(B)** Heat map of Spearman correlation analysis between 16 differential genera and 7 indicators. **(C)** Top 10 predicted metagenomic functions of the KEGG pathway at level 2 in feces. Red/blue represented positive/negative correlations, respectively, *P < 0.05, **P < 0.01, and ***P < 0.001.

To understand the intrinsic community interaction of 16 distinguishing genera, we employed a Spearman correlation analysis (Figure 3A) and the exact *P-*values were shown in Supplementary Table 2. Subsequently, a Spearman correlation analysis focused on 7 biological parameters and 16 genera was performed to determine the correlation between pneumonia and intestinal flora. Our results showed that *Facklamia* was positively correlated with IL-1β and MCP-1, and inversely correlated with SOD. *Staphylococcus* and *Jeotgalicoccus* were positively correlated with MCP-1, MPO and TNF-α, and inversely correlated with SOD. *Corynebacterium_1* was positively correlated with NO and MPO, and inversely correlated with SOD. Meanwhile, *Ruminiclostridium_1*, *Paenalcaligenes* and *Mucispirillum* were positively correlated with NO, and inversely correlated with IL-10 and SOD. Additionally, *Globicatella* was inversely correlated with NO and MPO. Interestingly, the majority of the genera abundant in the Model group were positively and inversely correlated with pro-inflammatory and anti-inflammatory factors, respectively (Figure 3B), and the *P-*values were exhibited in Supplementary Table 3. Moreover, PICRUSt analysis is conducive to understanding microbial KEGG functional prediction in fecal samples. Eight pathways were associated with metabolism, and two pathways were associated with genetic information processing. Among the top 10 pathways, in comparison to the Control group, the Model group’s lipid metabolism was significantly higher, and QKL downregulated lipid metabolism (Figure 3C).

### 3.4. QKL induced changes in metabolomic profiles

**FIGURE 4:**
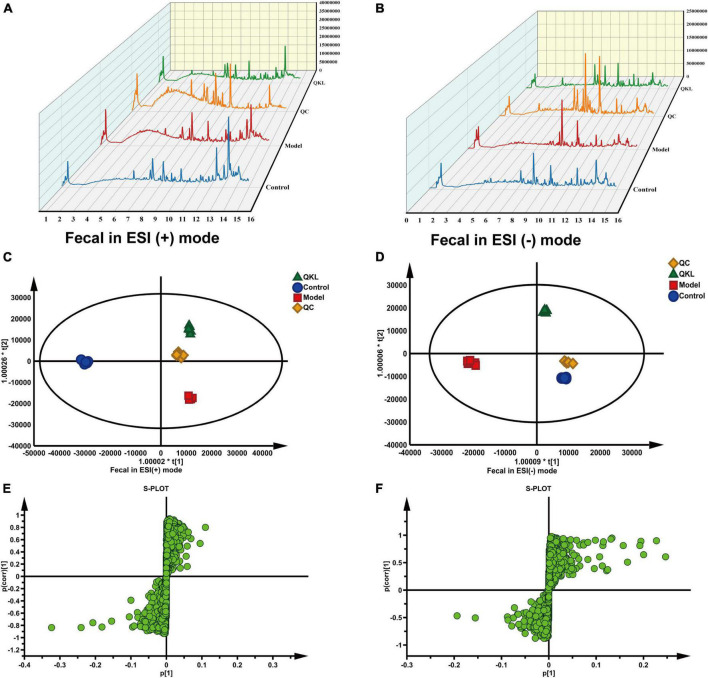
Effects of QKL on fecal metabolites in LPS-induced pneumonia. **(A,B)** Liquid chromatogram in fecal samples under ESI+ and ESI– modes, respectively. **(C,D)** OPLS-DA plot among different groups under two ESI modes. **(E,F)** S-plot between the Model and QKL groups under ESI+ and ESI– modes, respectively.

**FIGURE 5:**
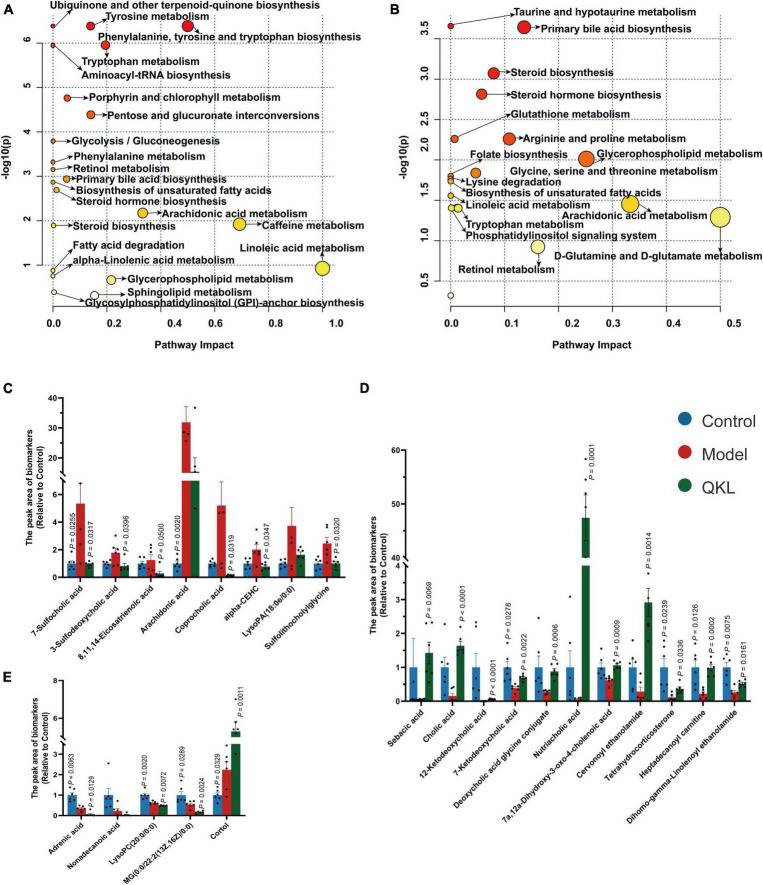
Altered host-microbiota co-metabolites among Control, Model and QKL groups. **(A,B)** Metabolic pathway analysis based on discriminant fecal metabolites using MetaboAnalyst 5.0. in the Control group compared with the Model group, the QKL group compared with the Model group, respectively. **(C–E)** Histogram of differential compounds.

A satisfying chromatographic peak shape occurred in ESI+ mode and ESI− mode (Figures 4A, B). Meanwhile, SIMCA v14.1 was used to perform multivariate statistical analysis to better understand the metabolite fingerprints of feces. The OPLS-DA score plot displayed great separation among distinct groups and desirable clustering of each group (Figures 4C, D), which indicated marked metabolic profile alterations in fecal samples under ESI+ mode (R^2^Y = 0.989, *Q*^2^ = 0.795) and ESI− mode (R^2^Y = 0.994, *Q*^2^ = 0.782). Furthermore, permutation tests (*n* = 200) were employed to avoid overfitting the model, and validation was performed in both ESI+ mode (R^2^Y = 0.836, *Q*^2^ = −0.685) and ESI– mode (R^2^Y = 0.767, *Q*^2^ = −0.599). The results showed that the OPLS-DA models were effective and did not overfit. In the OPLS-DA model, 1,861 (ESI−) and 1,642 (ESI+) variables between the Control and Model groups, and 1,511 (ESI−) and 1,095 (ESI+) variables between the Model and QKL groups were screened based on the S-plot (Figures 4E, F). Furthermore, according to the selection criteria (VIP > 1 and *P* < 0.05), 88 metabolites and 76 molecules were identified as potential metabolic biomarkers between the Control and Model groups (Supplementary Tables 4, 6), and the Model and QKL groups (Supplementary Tables 5, 6), respectively. Moreover, the differential metabolites were most abundant in carboxylic acids and derivatives, fatty acyls, indoles and derivatives, and other small molecules (Supplementary Tables 4–6), suggesting a large-scale metabolite dysregulation. To obtain the essential metabolic pathways, 88 and 76 differentially metabolites were imported into MetaboAnalyst V5.0. Consequently, our findings suggested that QKL treatment has a protective effect against LPS-induced inflammation in rats. This effect may be mediated, at least in part, by the regulation of various metabolic pathways, including arachidonic acid metabolism, glycerophospholipid metabolism, primary bile acid synthesis, steroid hormone biosynthesis and tryptophan metabolism (Figures 5A, B). Furthermore, 24 common metabolites, including 11 steroids and steroid derivatives, 7 fatty acyls, 3 glycerophospholipids, and 3 others were observed among the three groups (Supplementary Table 6), and their peak areas were compared to evaluate the protective effect after QKL therapy. As a result, the study demonstrated that compared with the Control group, the 24 metabolic compounds were altered by LPS administration and subsequently improved following treatment with QKL (Figures 5C–E). The 24 metabolites were involved in arachidonic acid metabolism, i.e., 8,11,14-eicosatrienoic acid (known as dihomo-γ-linolenic acid, DGLA) and arachidonic acid (AA); glycerophospholipid metabolism, i.e., LysoPC (20:0/0:0) and LysoPA (18:0e/0:0); and primary bile acid synthesis pathway, i.e., cholic acid. Collectively, these altered bioactive molecules may work together to reveal the therapeutic effects of QKL treatment.

### 3.5. Correlation analysis among fecal metabolites, intestinal flora, and inflammatory indices

**FIGURE 6:**
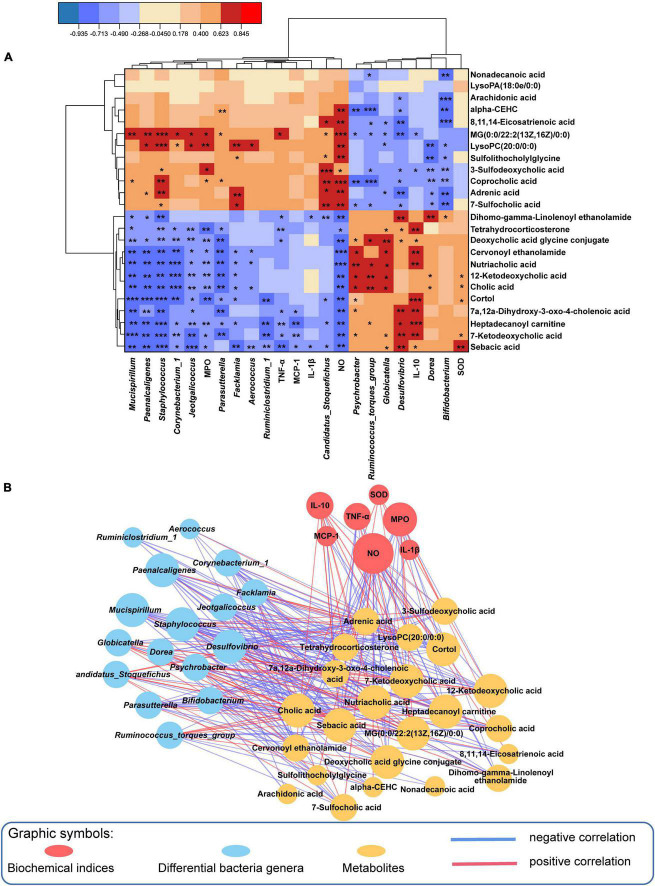
Spearman correlation analysis among the microbiome, biochemical indices and metabolome. **(A)** The results of 24 metabolites reversed by QKL administration were significantly correlated with 16 differential genera and some biochemical parameters using a Spearman correlation analysis, Red/blue represented positive/negative correlations, respectively, *P < 0.05, **P < 0.01, and ***P < 0.001. **(B)** Spearman correlation network (Spearman coefficient > 0.5 and *P* < 0.05). Red/blue lines indicated positive/negative correlations, respectively, and line thickness represented the correlation value.

To better determine the interaction of inflammatory parameters, 16 gut genera and 24 related metabolites, a Spearman correlation analysis was employed (Figures 6A, B), and Supplementary Table 7 displayed the *P-*values. Nutriacholic acid, cholic acid and 12-ketodeoxycholic acid were positively correlated with *Globicatella*, *Ruminococcus_torques_group* and *Psychrobacter*, and markedly inversely correlated with *Mucispirillum*, *Corynebacterium_1*, *Jeotgalicoccus*, *Staphylococcus*, *Facklamia*, and *Aerococcus*. In addition, the three metabolites were positively associated with SOD, and inversely associated with NO and MPO. AA, DGLA and αlpha-CEHC were significantly inversely correlated with *Bifidobacterium*, and positively associated with NO except AA. LysoPC (20:0/0:0) was markedly inversely correlated with *Bifidobacterium* and *Dorea*, and positively correlated with *Staphylococcus*, *Corynebacterium_1*, *Jeotgalicoccus* and *Aerococcus*, MG [0:0/22:2(13Z,16Z)/0:0] showed a similar correlation with LysoPC (20:0/0:0). In addition, LysoPC (20:0/0:0) was significantly positively associated with NO and MPO. In summary, the results demonstrated an evident and robust association between mostly gut genera and fecal metabolites, indicating that the modulation of specific metabolites was associated with enormous alterations in intestinal flora composition.

### 3.6. QKL regulated lung gene expression in rats with pneumonia

**FIGURE 7:**
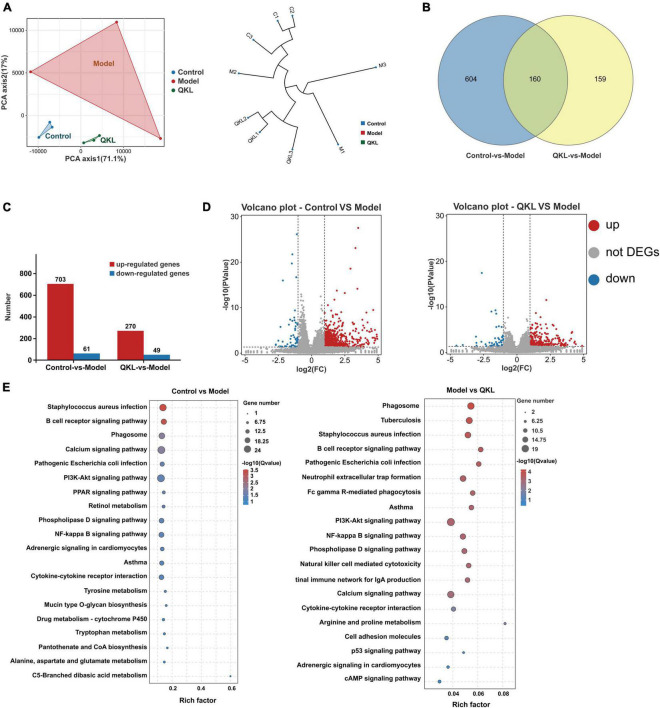
Altered differential genes (DEGs) after QKL therapy. **(A)** Principal component analysis and hierarchical clustering based on gene expression profiles. **(B)** Venn diagrams of DEGs in the three comparisons. **(C)** Up-regulated/down-regulated DEGs in the three comparisons. **(D)** Volcano plots show the DEGs from the comparison of Control vs. Model, Model VS QKL, respectively. **(E)** KEGG enrichment analysis of the DEGs between the Control vs. Model groups and the Model vs. QKL groups, respectively.

The results of transcriptome analysis revealed that QKL treatment significantly altered the gene expression profile in rats with LPS-induced pneumonia. The Q20 and Q30 were more than 97.23 and 92.40%, respectively, standing for the reliability of the transcriptome data. The PCA plot and hierarchical clustering demonstrated a significant cluster among the three groups. In addition, the PCA axis 1 and PCA axis 2 together explained 88.1% of the variance (Figure 7A). Then, 923 DEGs were discovered from the comparisons of Control vs. Model, and Model vs. QKL, with the cutoff of log2 (| fold change |) ≥ 2 and *P* < 0.05 (Figure 7B), using the Deseq2 package to analyze the DEGs. Among these, Control vs. Model and Model vs. QKL were screened, yielding 764 and 319 DEGs, respectively. Compared with the Control group, 61 and 703 genes were down-regulated and up-regulated in the Model group, respectively. In contrast, the QKL group generated 49 up-regulated and 270 down-regulated genes when compared with the Model group (Figures 7C, D). These gene alterations also indicated that a distinct gene expression pattern was induced after QKL treatment. In addition, the discriminated metabolic pathways of the 923 DEGs were found to be involved in 29 pathways (*P* < 0.05), including pathogenic *Escherichia coli* infection, PI3K-Akt signaling pathway, NF-κB signaling pathway, cytokine-cytokine receptor interaction, etc. (Figure 7E). Several pathways are known to be crucial in the pathogenesis of inflammation and pneumonia, and their modulation by QKL treatment may contribute to therapeutic effects.

### 3.7. Protein-protein network analysis of differentially expressed genes

**FIGURE 8:**
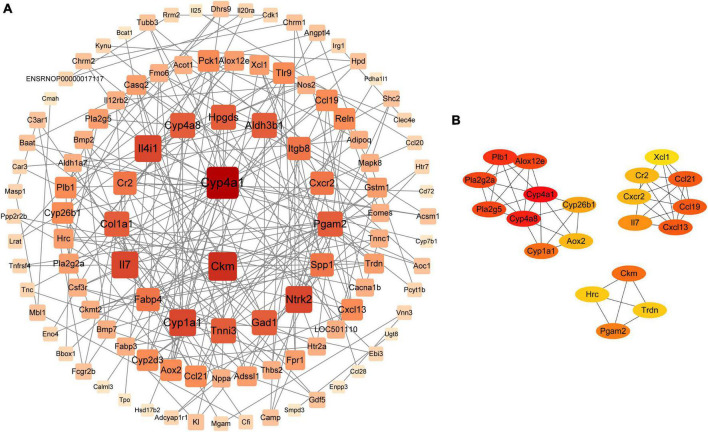
**(A)** PPI network of DEGs. **(B)** Top 20 core genes obtained in cytoHubba.

PPI network analysis was used to understand the correlation between DEGs. We gave priority attention to genes associated with 29 metabolic pathways. In total, 127 DEGs were obtained and imported to STRING,^4^ where they were visualized using Cytoscape V3.9.1. In addition, we deleted 18 genes that did not correlate with the 109 DEGs. PPI network analysis revealed that the top 20 core genes were highly associated with the DEGs identified in our study (Figure 8A). The maximal clique centrality (MCC) algorithm in the cytoHubba module was employed to further find the core genes. Consequently, the top 20 genes appeared: *Cxcl13*, *Ccl19*, *Ccl21*, *Cxcr2*, *Cr2*, *Il7*, *Xcl1*, *Pla2g2a*, *Pla2g5*, *Alox12e*, *Plb1*, *Cyp4a1*, *Cyp4a8*, *Cyp26b1*, *Aox2*, *Cyp1a1*, *Ckm*, *Hrc*, *Trdn*, and *Pgam2* (Figure 8B), and a heat map of 20 genes was depicted (Supplementary Figure 3). Moreover, the 20 genes were involved in various metabolic pathways, including arachidonic acid metabolism and cytokine-cytokine receptor interaction, etc. Interestingly, some of these pathways were also enriched in the microbiome and metabolome analyses, suggesting a potential microbiota-metabolites-host interaction. Specifically, *Il7* was associated with the PI3K-Akt signaling pathway. *Il7*, *Cr2*, *Xcl1* and chemokine-related genes (e.g., *Cxcl13*, *Ccl19*, *Ccl21*, and *Cxcr2*), all of which were involved in cytokine-cytokine receptor interaction. *Ccl19* and *Ccl21* were also related to the NF-κB signaling pathway. *Pla2g2a*, *Pla2g5*, *Alox12e*, *Plb1*, *Cyp4a1*, and *Cyp4a8* were related to arachidonic acid metabolism. *Cyp26b1*, *Aox2*, and *Cyp1a1* were involved in retinol metabolism. In addition, *Aox2* and *Cyp1a1* were associated with tryptophan metabolism.

## 4. Discussion

While the pathogenesis of pneumonia is not fully understood, recent studies have shown a link between the gastrointestinal and respiratory tracts, suggesting that pneumonia may be associated with intestinal flora disorders. Intestinal flora dysbiosis is found in mice with Streptococcus pneumoniae-induced pneumonia (Feng et al., 2020). In addition, intestinal flora produces some dispersal and specific metabolites that enter the systemic circulation and have distal effects including on the lung (Steed et al., 2017). Several metabolomics investigations have elaborated on wide disturbances of metabolites related to intestinal flora dysbiosis in pneumonia (de Vos et al., 2022; Huo et al., 2022). Some studies have reported the protective effect of CMs on pneumonia by regulating intestinal flora disorders and metabolite abnormalities (Lian et al., 2022; Wu et al., 2022). While single omics technologies such as the microbiome, metabolome, and transcriptome have been extensively applied in current studies, it is important to note that each of these technologies reflects only one aspect of the complex interactions between the host, microbiota, and environment (Tian et al., 2022). Multiple omics are beneficial in systematically elucidating the pathogenic mechanism of pneumonia and obtaining key biomarkers for diagnosing and treating pneumonia. Owing to the sophistication of CMs, elucidating the protective mechanism is more challenging. Rarely has the mechanism of the anti-pneumonic effect of QKL oral liquid, a classic recipe for respiratory disease, been reported from the perspective of intestinal flora and host-microbiota co-metabolites. In the study, we evaluated the therapeutic effects of QKL treatment on LPS-induced pneumonia rats and explored the mechanism using an integrated analysis of the fecal microbiome, gut metabolome and lung transcriptome.

Intestinal flora is closely associated with the occurrence and progression of pneumonia (Ma et al., 2022). Animals with a lower ratio of F/B prevent allergic lung inflammation (Trompette et al., 2014). The proportion of F/B in the QKL group was also markedly reduced in the study. Bacteroidetes, can offer nutrition to the host and other intestinal flora by metabolizing indigestible saccharides (Zafar and Saier, 2021), which may contribute to preserving intestinal homeostasis. Moreover, QKL treatment increased the abundance of *Bifidobacterium*, *Dorea* and *Ruminococcus_torques_group*, etc. Treatment with *Bifidobacterium longum* subsp. *longum* prevents the mice from smoke-induced lung inflammation by decreasing the expression of inflammatory cytokines and adhesion factors (Budden et al., 2022). In addition, *Bifidobacterium* and *Dorea* are members of short-chain fatty acids (SCFAs)-generating bacteria. Furthermore, SCFAs can maintain intestinal barrier integrity and thus inhibit the translocation of host-microbiota co-metabolites and inflammatory mediators from the gut into distal organs, including lung (Upadhyaya and Banerjee, 2015; Fernandez et al., 2020). As a result, the increase in *Bifidobacterium* and *Dorea* may be associated with the protective effect of QKL on intestinal mucosal barrier, which inhibits pathogens translocation. *Ruminococcus_torques_group* is widely regarded as beneficial bacteria that improve immunity (Bai et al., 2022). Interestingly, QKL treatment also decreased the abundance of *Mucispirillum*, *Staphylococcus*, *Corynebacterium_1*, etc. *Mucispirillum*, serving as a mucin degrader, damages the intestinal integrity (Berry et al., 2012). The reduction in *Mucispirillum* after QKL treatment might have beneficial effects against LPS-stimulated lower integrity, reducing intestinal leakage. *Staphylococcus* intrudes into the host in several ways and induces local and systemic infections including pneumonia (Ranzani et al., 2020). In addition, the opportunistic pathogen *Staphylococcus aureus* can generate staphylococcal enterotoxin B, and thus induce lung inflammatory cell infiltration and lung lesions (Zong et al., 2022). Taken together, these results indicated that QKL could regulate intestinal flora dysbiosis, especially upregulating the abundance of *Bifidobacterium* and *Dorea*, but downregulating the abundance of *Mucispirillum* and *Staphylococcus*, which might be responsible for the maintenance of intestinal integrity, inhibition of pathogens translocation and suppression of inflammatory cytokines secretion, causing the mitigation of pneumonia.

Metabolite dysfunction is a leading cause in the pathogenesis of pneumonia as well. Several findings have proven that metabolic profile alterations and metabolic disturbances are observed in pneumonia patients (Li et al., 2022). Furthermore, fatty acyls, glycerophospholipids and sphingolipids were significantly altered metabolites, and they were involved in arachidonic acid metabolism, glycerophospholipid metabolism, steroid hormone biosynthesis and tryptophan metabolism in the study. AA and DGLA are fatty acyls and unsaturated fatty acids, generating inflammatory mediators such as a series of prostaglandins and leukotrienes and thereby inducing inflammation (Belch and Hill, 2000; Perreault et al., 2014; Wang et al., 2019). AA administration alters the structure of intestinal flora and damages the intestinal barrier, causing multi-organ inflammation in high-fat diet mice (Zhuang et al., 2017). Although some environmental microorganisms, such as fungi (*Mortierella*, *Diasporangium*, and *Pythium*) and bacteria (*Aureispira maritime*), can produce AA, the effects of intestinal flora on AA production remain unknown (Fu et al., 2023). In addition to fatty acids, glycerophospholipids, consisting of LysoPC, LysoPA, PC, and PE in the study, were significantly restored after QKL treatment. Specifically, LysoPC and LysoPA, belonging to the lysophosphatidylcholine (LPC) family, are critical signaling molecules in multiple bioactive metabolisms (Wu et al., 2022). Moreover, LPC can promote the generation of pro-inflammatory factors (Liu et al., 2020). For LysoPC, there is an indirect shift toward the formation of platelet-activating factor, thereby inducing inflammation (Gill et al., 2015). In the study, treatment with QKL prevented pneumonia in rats, which may be due to the reduction in the levels of pro-inflammatory mediators (e.g., AA and DGLA) and glycerophospholipids. Moreover, the study’s Spearman correlation analysis indicated that the contents of AA, DGLA, and LysoPC (20:0/0:0) were inversely correlated with the relative abundance of *Bifidobacterium*, suggesting the potential effect of *Bifidobacterium* on host arachidonic acid and glycerophospholipid metabolism. Steroids and steroid derivatives were also significantly altered, especially bile acids, which belong to steroid acids. They are synthesized in the liver and mainly secreted into the gut, involving multiple bioactive processes including cholesterol, glucose and energy homeostasis (Perino and Schoonjans, 2022). The contents of host-derived primary BAs (e.g., cholic acid (CA) and nutriacholic acid) and microbiota-derived secondary BAs (e.g., 7-ketodeoxycholic acid and 12-ketodeoxycholic acid) were elevated after QKL treatment. CA can activate the farnesoid X receptor, thus maintaining intestinal flora homeostasis, preventing bacterial translocation, and enhancing mucosal barrier defense (Staley et al., 2017). Furthermore, the generation and diversity of BAs are associated with host and microbial metabolism (Perino and Schoonjans, 2022). For instance, genera such as *Bacteroides*, *Clostridium*, *Lactobacillus*, *Bifidobacterium*, and *Ruminococcus* can convert the primary BAs to diverse BAs using bile salt hydrolases (Staley et al., 2017; Winston and Theriot, 2020). In the study, the genera producing secondary BAs were partially increased after QKL treatment, including *Bacteroides*, *Ruminococcus_torques_group* and *Bifidobacterium*. These genera may work together with the host by regulating BAs to improve pneumonia. Furthermore, after treatment with QKL, a Spearman correlation analysis demonstrated a holistic correlation among the intestinal flora, host-microbiota co-metabolites and inflammatory indices. The analysis also showed that QKL treatment could mitigate LPS-induced pneumonia by reshaping the intestinal flora and host-microbiota co-metabolites.

Interestingly, the KEGG analysis of the fecal metabolome and lung transcriptome showed that QKL could modulate lipid metabolism, especially arachidonic acid metabolism, which coincided with the results of microbiome. The study showed that the core 20 DEGs were involved in arachidonic acid metabolism and inflammation-related pathways (e.g., NF-κB signal pathway). Further study revealed that the expression of genes (*Pla2g2a*, *Pla2g5*, *Alox12e*, and *Cyp4a8*) involved with arachidonic acid metabolism was increased in the Model group. Specifically, *Pla2g2a* and *Pla2g5* genes, which are members of secretory phospholipase A2 family, can encode enzymes that liberate membrane phospholipids to produce free fatty acids such as AA (Brant et al., 2006). In addition, phospholipase A2 activity is increased under inflammation, causing elevated *Alox12e* gene expression, and the expression of pro-inflammatory cytokines is decreased after inhibition of *Alox12e* (Srikanth et al., 2018). In brief, QKL treatment probably reduced the gene (*Pla2g2a*, *Pla2g5*, and *Alox12e*) expression and AA levels to alleviate pneumonia. *Ccl19* and *Ccl21* genes are involved in modulating NF-κB pathway activation, and NF-κB signaling pathway is activated in LPS-induced lung injury animals. Furthermore, inhibiting the pathway can alleviate lung inflammation (Tang et al., 2021). Moreover, *Ccl19* expression is associated with *Staphylococcus*. When *Staphylococcus aureus* is given to mice with pneumonia, enterotoxin B is released, which raises *Ccl19* levels in lungs and worsens lung inflammation (Huvenne et al., 2011). In the study, the abundance of *Staphylococcus* rose, which might upregulate *Ccl19* expression and exacerbate pneumonia, and QKL reversed these effects. Collectively, the therapeutic effects of QKL treatment on alleviating pneumonia may be dependent on suppression of the NF-κB pathway, reduction of gene (*Ccl19* and *Ccl21*) expression and *Staphylococcus* abundance. Indole-3-carboxaldehyde level was increased in the QKL group compared with the Model group (Supplementary Table 5). Indole-3-carboxaldehyde may promote aryl hydrocarbon receptor (AhR) activation (Roager and Licht, 2018), inducing *Cyp1a1* overexpression (Fujii Kuriyama and Mimura, 2005). A high level of *Cyp1a1* mRNA is vital to maintain intestinal barrier integrity (Ghosh et al., 2022). Moreover, *Cyp1a1*^–/–^ mice displayed elevated susceptibility to oxygen-induced lung damage and inflammation (Maturu et al., 2017). The study found that QKL treatment led to an increase in the mRNA expression of *Cyp1a1*, which suggests that this may be one of the mechanisms by which QKL helps to prevent pneumonia.

**FIGURE 9:**
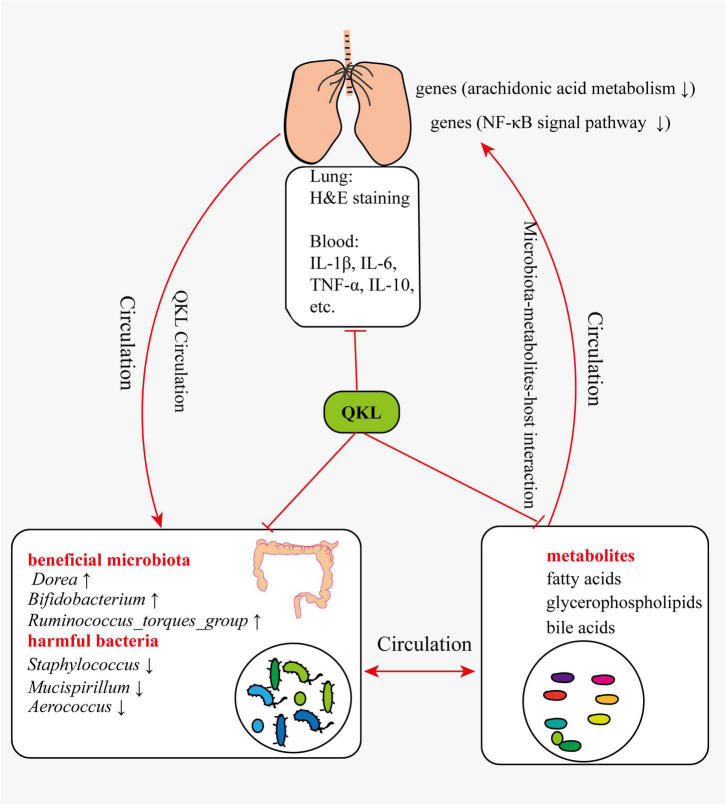
Potential mechanism of QKL treatment in attenuating pneumonia.

In summary, pneumonia symptoms were alleviated following treatment with QKL, and the study provided a comprehensive understanding of the mechanism by which QKL treatment on pneumonia. The multi-omics technologies enabled us to elucidate the complex interaction among intestinal flora, host-microbiota co-metabolits and host. Based on the results of the study and references, we suggested the following perspectives: (1) QKL treatment regulated LPS-induced intestinal flora dysbiosis, including significantly increasing the abundance of beneficial microbiota (e.g., *Bifidobacterium*, *Ruminococcus_torques_group* and *Dorea*) and depleting the abundance of harmful bacteria (e.g., *Staphylococcus*, *Mucispirillum* and *Aerococcus*). (2) QKL treatment protected the rats against pneumonia by regulating various metabolic pathways, including arachidonic acid metabolism, glycerophospholipid metabolism and inflammation-related pathways. Intestinal flora could also be involved in metabolic processes, and differential metabolites could affect the structure of intestinal microbiota. (3) QKL treatment exerted its therapeutic effects on rats with pneumonia by regulating lung gene (*Pla2g2a*, *Pla2g5*, *Alox12e*, *Cyp4a8*, *Ccl19*, and *Ccl21*) expression associated with arachidonic acid metabolism and inflammation-related pathways (e.g., NF-κB signal pathway). The probable mechanism was shown in the diagrammatic drawing (Figure 9). Although we proposed possible reasonings according to the current results, there are still some limitations. For instance, these were a relatively small number of samples in the study, and subsequent investigations employing larger sample sizes are imperative to corroborate the present findings. Second, verifying these perspectives using fecal microbiota transplantation and core gene knockout experiments to further understand the roles of the identified biomarkers in alleviating pneumonia is required. Third, the study showed a strong association on multi-omics results without demonstrating causality or directionality.

## 5. Conclusion

Pneumonia symptoms were alleviated following treatment with QKL, and this could potentially be attributed to modulation of intestinal flora structure, host-microbiota co-metabolites composition and lung gene expression, providing fresh ideas on the microbiota-metabolites-host interaction and clinical therapy in QKL mitigating pneumonia.

## Data availability statement

The datasets presented in this study can be found in online repositories. The names of the repository/repositories and accession number(s) can be found in the article/Supplementary material.

## Ethics statement

This animal study was reviewed and approved by the Institutional Animal Care and Ethics Committee of Sun Yat-sen University.

## Author contributions

HC and SL performed all experiments and wrote the first draft. BP managed sample collection and carried out the experiment. KL and HY assisted in analysis and revised the manuscript. CM, HQ, and YZ revised the manuscript. ZX and DO offered economic support and revised the manuscript. All authors contributed to the article and approved the submitted version.

## Footnotes

1
https://www.hmdb.ca/

2
https://www.kegg.jp

3
https://www.metaboanalyst.ca/

4
https://cn.string-db.org//

## References

[B1] Bai Y. S. Ma K. D. Li J. B. Ren Z. S. Zhang J. Shan A. S. (2022). *Lactobacillus rhamnosus* GG ameliorates DON-induced intestinal damage depending on the enrichment of beneficial bacteria in weaned piglets. *J. Anim. Sci. Biotechnol*. 13:90. 10.1186/s40104-022-00737-9 35962456 PMC9375241

[B2] Belch J. J. Hill A. (2000). Evening primrose oil and borage oil in rheumatologic conditions. *Am. J. Clin. Nutr*. 71 352–356. 10.1093/ajcn/71.1.352s 10617996

[B3] Berry D. Schwab C. Milinovich G. Reichert J. Ben Mahfoudh K. Decker T. (2012). Phylotype-level 16S rRNA analysis reveals new bacterial indicators of health state in acute murine colitis. *ISME J*. 6 2091–2106. 10.1038/ismej.2012.39 22572638 PMC3475367

[B4] Brant K. Guan W. Tithof P. Caruso R. L. (2006). Gestation age-related increase in 50-kDa rat uterine calcium-independent phospholipase A2 expression influences uterine sensitivity to polychlorinated biphenyl stimulation. *Biol. Reprod*. 74 874–880. 10.1095/biolreprod.105.047084 16436530

[B5] Budden K. F. Gellatly S. L. Vaughan A. Amorim N. Horvat J. C. Hansbro N. G. (2022). Probiotic *Bifidobacterium longum* subsp. *longum* protects against cigarette smoke-induced inflammation in mice. *Int. J. Mol. Sci*. 24:252. 10.3390/ijms24010252 36613693 PMC9820259

[B6] Chen H. Y. Li X. Xu J. Zhang T. J. Li Q. Ouyang D. S. (2022). Mechanism of Qingkailing oral liquid in preventing and treatment of pneumonia based on TLR4/NF-κB signaling pathway. *Chin. Tradit. Herb. Drugs* 53 6101–6107. 10.7501/j.issn.0253-2670.2022.19.016

[B7] Dang A. T. Marsland B. J. (2019). Microbes, metabolites, and the gut-lung axis. *Mucosal Immunol*. 12 843–850. 10.1038/s41385-019-0160-6 30976087

[B8] de Vos W. M. Tilg H. Van Hul M. Cani P. D. (2022). Gut microbiome and health: Mechanistic insights. *Gut* 71 1020–1032. 10.1136/gutjnl-2021-326789 35105664 PMC8995832

[B9] Deng L. Shi Y. C. Liu P. Wu S. Z. Lv Y. W. Xu H. C. (2021). GeGen QinLian decoction alleviate influenza virus infectious pneumonia through intestinal flora. *Biomed. Pharmacother*. 141:111896. 10.1016/j.biopha.2021.111896 34246956

[B10] Feng J. L. Dai W. B. Zhang C. Chen H. J. Chen Z. L. Chen Y. F. (2020). Shen-ling-bai-zhu-san ameliorates inflammation and lung injury by increasing the gut microbiota in the murine model of *Streptococcus pneumonia*-induced pneumonia. *BMC Complement. Med. Ther*. 20:159. 10.1186/s12906-020-02958-9 32460745 PMC7254717

[B11] Fernandez J. de la Fuente V. G. Garcia M. T. F. Sanchez J. G. Redondo B. I. Villar C. J. (2020). A diet based on cured acorn-fed ham with oleic acid content promotes anti-inflammatory gut microbiota and prevents ulcerative colitis in an animal model. *Lipids Health Dis*. 19:28. 10.1186/s12944-020-01205-x 32093685 PMC7041278

[B12] Fu J. F. Shan J. L. Cui Y. Z. Yan C. Z. Wang Q. Z. Han J. X. (2023). Metabolic disorder and intestinal microflora dysbiosis in chronic inflammatory demyelinating polyradiculoneuropathy. *Cell Biosci*. 13:6. 10.1186/s13578-023-00956-1 36627678 PMC9832664

[B13] Fujii Kuriyama Y. Mimura J. (2005). Molecular mechanisms of AhR functions in the regulation of cytochrome P450 genes. *Biochem. Biophys. Res. Commun*. 338 311–317. 10.1016/j.bbrc.2005.08.162 16153594

[B14] Ghosh S. Moorthy B. Haribabu B. Jala V. R. (2022). Cytochrome P450 1A1 is essential for the microbial metabolite, Urolithin A-mediated protection against colitis. *Front. Immunol*. 13:1004603. 10.3389/fimmu.2022.1004603 36159798 PMC9493474

[B15] Gill P. Jindal N. L. Jagdis A. Vadas P. (2015). Platelets in the immune response: Revisiting platelet-activating factor in anaphylaxis. *J. Allergy Clin. Immunol*. 135 1424–1432. 10.1016/j.jaci.2015.04.019 26051949

[B16] Huo J. J. Wang T. Wei B. K. Shi X. L. Yang A. D. Chen D. F. (2022). Integrated network pharmacology and intestinal flora analysis to determine the protective effect of Xuanbai-Chengqi decoction on lung and gut injuries in influenza virus-infected mice. *J. Ethnopharmacol*. 298:115649. 10.1016/j.jep.2022.115649 35987410

[B17] Huvenne W. Lanckacker E. A. Krysko O. Bracke K. R. Demoor T. Hellings P. W. (2011). Exacerbation of cigarette smoke induced pulmonary inflammation by *Staphylococcus aureus* enterotoxin B in mice. *Respir. Res*. 12:69. 10.1186/1465-9921-12-69 21615971 PMC3125222

[B18] Li C. X. Liu H. Y. Lin Y. X. Pan J. B. Su J. (2020). The gut microbiota and respiratory diseases: New evidence. *J. Immunol. Res*. 2020:2340670. 10.1155/2020/2340670 32802893 PMC7415116

[B19] Li J. Q. Luu L. D. W. Wang X. X. Cui X. D. Huang X. L. Fu J. (2022). Metabolomic analysis reveals potential biomarkers and the underlying pathogenesis involved in mycoplasma pneumoniae pneumonia. *Emerg. Microbes Infect*. 11 593–605. 10.1080/22221751.2022.2036582 35094669 PMC8865114

[B20] Lian B. He S. S. Jiang H. Guo Y. H. Cui X. R. Jiang T. (2022). Qin-Qiao-Xiao-Du formula alleviate influenza virus infectious pneumonia through regulation gut microbiota and metabolomics. *Front. Med. (Lausanne)* 9:1032127. 10.3389/fmed.2022.1032127 36313993 PMC9614278

[B21] Liu P. P. Zhu W. Chen C. Yan B. Zhu L. Chen X. (2020). The mechanisms of lysophosphatidylcholine in the development of diseases. *Life Sci*. 247:117443. 10.1016/j.lfs.2020.117443 32084434

[B22] Ma P. J. Wang M. M. Wang Y. (2022). Gut microbiota: A new insight into lung diseases. *Biomed. Pharmacother*. 155:113810. 10.1016/j.biopha.2022.113810 36271581

[B23] Manzanares W. Lemieux M. Langlois P. L. Wischmeyer P. E. (2016). Probiotic and synbiotic therapy in critical illness: A systematic review and meta-analysis. *Crit. Care* 19:262. 10.1186/s13054-016-1434-y 27538711 PMC4991010

[B24] Maturu P. Weiliang Y. H. Jiang W. W. Wang L. H. Lingappan K. Barrios R. (2017). Newborn mice lacking the gene for Cyp1a1 are more susceptible to oxygen-mediated lung injury, and are rescued by postnatal beta-naphthoflavone administration: Implications for bronchopulmonary dysplasia in premature infants. *Toxicol. Sci*. 157 260–271. 10.1093/toxsci/kfx036 28201809 PMC5837454

[B25] Peng L. Y. Shi H. T. Tan Y. R. Shen S. Y. Yi P. F. Shen H. Q. (2021). Baicalin inhibits APEC-induced lung injury by regulating gut microbiota and SCFA production. *Food Funct*. 12 12621–12633. 10.1039/d1fo02407h 34821232

[B26] Perino A. Schoonjans K. (2022). Metabolic messengers: Bile acids. *Nat. Metab*. 4 416–423. 10.1038/s42255-022-00559-z 35338368

[B27] Perreault M. Roke K. Badawi A. Nielsen D. E. Abdelmagid S. A. El-Sohemy A. (2014). Plasma levels of 14:0, 16:0, 16:1n-7, and 20:3n-6 are positively associated, but 18:0 and 18:2n-6 are inversely associated with markers of inflammation in young healthy adults. *Lipids* 49 255–263. 10.1007/s11745-013-3874-3 24338596

[B28] Ranzani O. T. Motos A. Chiurazzi C. Ceccato A. Rinaudo M. Li Bassi G. (2020). Diagnostic accuracy of gram staining when predicting staphylococcal hospital-acquired pneumonia and ventilator-associated pneumonia: A systematic review and meta-analysis. *Clin. Microbiol. Infect*. 26 1456–1463. 10.1016/j.cmi.2020.08.015 32822880

[B29] Roager H. M. Licht T. R. (2018). Microbial tryptophan catabolites in health and disease. *Nat. Commun*. 9:3294. 10.1038/s41467-018-05470-4 30120222 PMC6098093

[B30] Rotta A. T. Gunnarsson B. Hernan L. J. Fuhrman B. P. Steinhorn D. M. (1999). Partial liquid ventilation influences pulmonary histopathology in an animal model of acute lung injury. *J. Crit. Care* 14 84–92. 10.1016/S0883-9441(99)90019-9 10382789

[B31] Srikanth M. Chandrasaharan K. Zhao X. Chayaburakul K. Ong W. Y. Herr D. R. (2018). Metabolism of docosahexaenoic acid (DHA) induces pyroptosis in BV-2 microglial cells. *Neuromolecular Med*. 20 504–514. 10.1007/s12017-018-8511-0 30232677

[B32] Staley C. Weingarden A. R. Khoruts A. Sadowsky M. J. (2017). Interaction of gut microbiota with bile acid metabolism and its influence on disease states. *Appl. Microbiol. Biotechnol*. 101 47–64. 10.1007/s00253-016-8006-6 27888332 PMC5203956

[B33] Steed A. L. Christophi G. P. Kaiko G. E. Sun L. L. Goodwin V. M. Jain U. (2017). The microbial metabolite desaminotyrosine protects from influenza through type I interferon. *Science* 357 498–502. 10.1126/science.aam5336 28774928 PMC5753406

[B34] Synodinou K. D. Nikolaki M. D. Triantafyllou K. Kasti A. N. (2022). Immunomodulatory effects of probiotics on COVID-19 infection by targeting the gut-lung axis microbial cross-talk. *Microorganisms* 10:1764. 10.3390/microorganisms10091764 36144365 PMC9505869

[B35] Tang J. Xu L. Q. Zeng Y. W. Gong F. (2021). Effect of gut microbiota on LPS-induced acute lung injury by regulating the TLR4/NF-κB signaling pathway. *Int. Immunopharmacol*. 91:107272. 10.1016/j.intimp.2020.107272 33360370

[B36] Tian H. C. Wen Z. Y. Liu Z. C. Guo Y. Q. Liu G. B. Sun B. L. (2022). Comprehensive analysis of microbiome, metabolome and transcriptome revealed the mechanisms of *Moringa oleifera* polysaccharide on preventing ulcerative colitis. *Int. J. Biol. Macromol*. 222 573–586. 10.1016/j.ijbiomac.2022.09.100 36115453

[B37] Torres A. Cilloniz C. Niederman M. S. Menendez R. Chalmers J. D. Wunderink R. G. (2021). Pneumonia. *Nat. Rev. Dis. Primers* 7:25. 10.1038/s41572-021-00259-0 33833230

[B38] Trompette A. Gollwitzer E. S. Yadava K. Sichelstiel A. K. Sprenger N. Ngom-Bru C. (2014). Gut microbiota metabolism of dietary fiber influences allergic airway disease and hematopoiesis. *Nat. Med*. 20 159–166. 10.1038/nm.3444 24390308

[B39] Upadhyaya S. Banerjee G. (2015). Type 2 diabetes and gut microbiome: At the intersection of known and unknown. *Gut Microbes* 6 85–92. 10.1080/19490976.2015.1024918 25901889 PMC4615359

[B40] Wan F. Zhong R. Q. Wang M. Y. Zhou Y. X. Chen Y. X. Yi B. (2021). Caffeic acid supplement alleviates colonic inflammation and oxidative stress potentially through improved gut microbiota community in mice. *Front. Microbiol*. 12:784211. 10.3389/fmicb.2021.784211 34867926 PMC8636926

[B41] Wang T. Q. Fu X. J. Chen Q. F. Patra J. K. Wang D. D. Wang Z. G. (2019). Arachidonic acid metabolism and kidney inflammation. *Int. J. Mol. Sci*. 20:3683. 10.3390/ijms20153683 31357612 PMC6695795

[B42] Winston J. A. Theriot C. M. (2020). Diversification of host bile acids by members of the gut microbiota. *Gut Microbes* 11 158–171. 10.1080/19490976.2019.1674124 31595814 PMC7053883

[B43] Wu G. S. Zhang W. D. Zheng N. N. Zu X. P. Tian S. S. Zhong J. (2022). Integrated microbiome and metabolome analysis reveals the potential therapeutic mechanism of Qing-Fei-Pai-Du decoction in mice with coronavirus-induced pneumonia. *Front. Cell. Infect. Microbiol*. 12:950983. 10.3389/fcimb.2022.950983 36093201 PMC9461713

[B44] Yang Q. Y. Ma L. L. Zhang C. Lin J. Z. Han L. He Y. N. (2021). Exploring the mechanism of indigo naturalis in the treatment of uicerative colitis based on TLR4/MyD88/NF-kappaB signaling pathway and gut microbiota. *Front. Pharmacol*. 12:674416. 10.3389/fphar.2021.674416 34366843 PMC8339204

[B45] Zafar H. Saier M. H. Jr. (2021). Gut *bacteroides* species in health and disease. *Gut Microbes* 13 1–20. 10.1080/19490976.2020.1848158 33535896 PMC7872030

[B46] Zeng J. Wang C. T. Zhang F. S. Qi F. Wang S. F. Ma S. (2016). Effect of probiotics on the incidence of ventilator-associated pneumonia in critically ill patients: A randomized controlled multicenter trial. *Intensive Care Med*. 42 1018–1028. 10.1007/s00134-016-4303-x 27043237

[B47] Zhang D. Li S. Wang N. Tan H. Y. Zhang Z. Feng Y. (2020). The cross-talk between gut microbiota and lungs in common lung diseases. *Front. Microbiol*. 11:301. 10.3389/fmicb.2020.00301 32158441 PMC7052046

[B48] Zhang H. Y. Hu P. Luo G. A. Liang Q. L. Wang Y. L. Yan S. K. (2006). Screening and identification of multi-component in Qingkailing injection using combination of liquid chromatography/time-of-flight mass spectrometry and liquid chromatography/ion trap mass spectrometry. *Anal. Chim. Acta* 577 190–200. 10.1016/j.aca.2006.06.053 17723671

[B49] Zhang Y. M. Li Y. Sun N. Tang H. Q. Ye J. Liu Y. (2022). NETosis is critical in patients with severe community-acquired pneumonia. *Front. Immunol*. 13:1051140. 10.3389/fimmu.2022.1051140 36466920 PMC9709478

[B50] Zhang Y. R. Gan Z. Q. Liu Z. X. Luo J. Tang C. Liu C. (2020). Exploring mechanism of Qingkailing injection in treatment of coronavirus disease 2019 (COVID-19) based on network pharmacology and molecular docking. *Chin. Tradit. Herb. Drugs* 51 3201–3210. 10.7501/j.issn.0253-2670.2020.12.012

[B51] Zhang Z. Wu X. Y. Cao S. Y. Cromie M. Shen Y. H. Feng Y. M. (2017). Chlorogenic acid ameliorates experimental colitis by promoting growth of Akkermansia in mice. *Nutrients* 9:677. 10.3390/nu9070677 28661454 PMC5537792

[B52] Zhou F. Liu B. Liu X. Li Y. Wang L. Huang J. (2021). The impact of microbiome and microbiota-derived sodium butyrate on *Drosophila* transcriptome and metabolome revealed by multi-omics analysis. *Metabolites* 11:298. 10.3390/metabo11050298 34066348 PMC8148185

[B53] Zhuang P. Shou Q. Y. Lu Y. H. Wang G. F. Qiu J. N. Wang J. (2017). Arachidonic acid sex-dependently affects obesity through linking gut microbiota-driven inflammation to hypothalamus-adipose-liver axis. *Biochim. Biophys. Acta Mol. Basis Dis*. 1863 2715–2726. 10.1016/j.bbadis.2017.07.003 28711599

[B54] Zong F. L. Gan C. J. Wang Y. F. Su D. Deng M. Y. Xiao N. (2022). Exposure to aerosolized staphylococcal enterotoxin B potentiated by lipopolysaccharide modifies lung transcriptomes and results in lung injury in the mouse model. *J. Appl. Toxicol*. 42 1205–1217. 10.1002/jat.4289 35080034

[B55] Zuo T. Zhang F. Lui G. C. Y. Yeoh Y. K. Li A. Y. L. Zhan H. (2020). Alterations in gut microbiota of patients with COVID-19 during time of hospitalization. *Gastroenterology* 159 944–955. 10.1053/j.gastro.2020.05.048 32442562 PMC7237927

