# Supplementary material for: Qing-Kai-Ling oral liquid alleviated pneumonia via regulation of intestinal flora and metabolites in rats
Source: Front Microbiol. 2023 Jun 9;14:1194401. doi: 10.3389/fmicb.2023.1194401 (PMC10288885; doi:10.3389/fmicb.2023.1194401)
Supplement: Supplementary file 1 [file Data_Sheet_1.PDF]

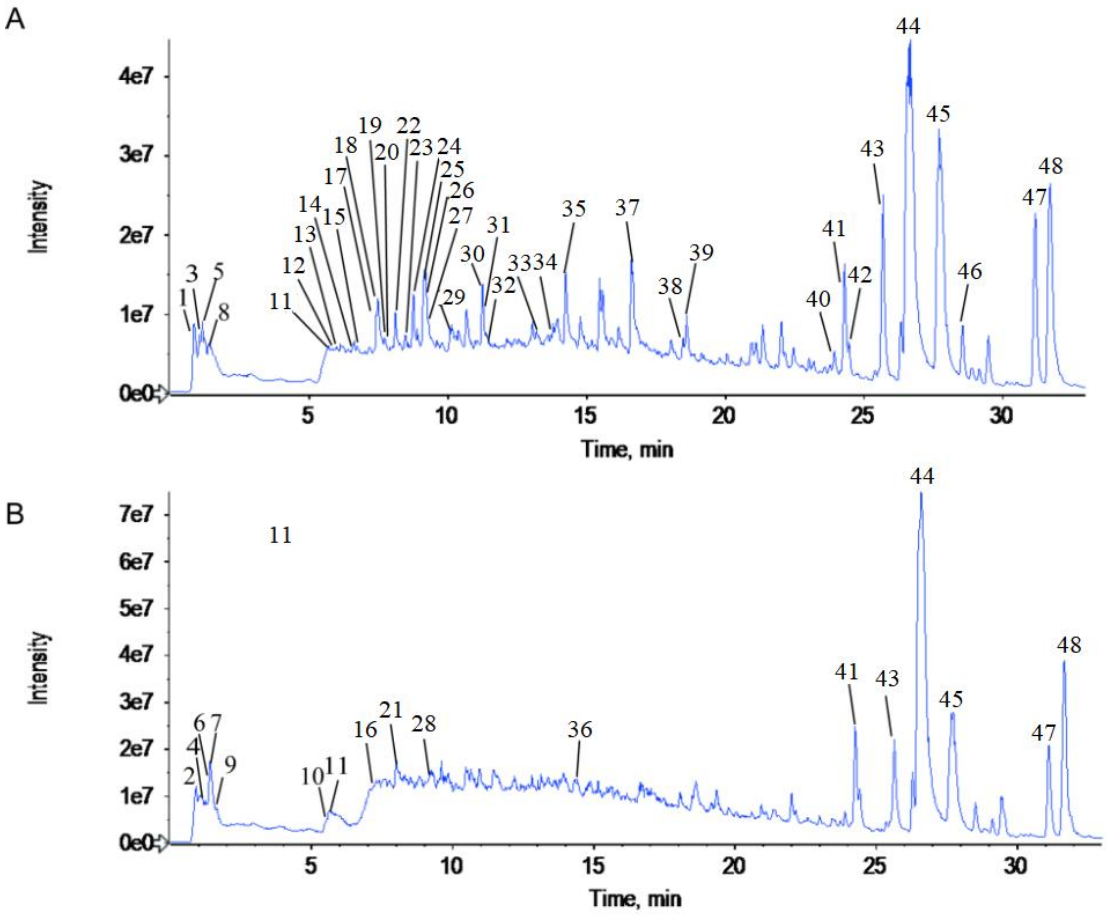

Supplementary Figure 1

Fig. 1. Total ion chromatogram (TIC) of Qing-Kai-Ling oral liquid in ESI- (A) ion and mode ESI+ (B), respectively.

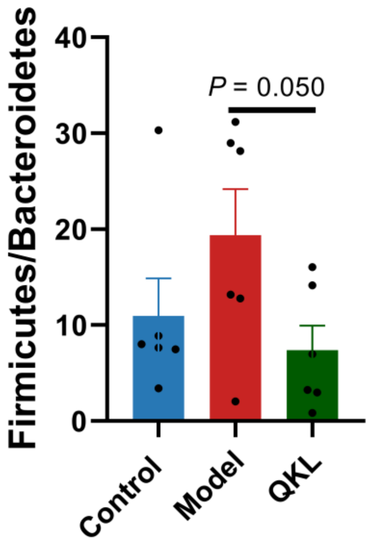

Supplementary Figure 2

Ratio of Firmicutes/Bacteroidetes.

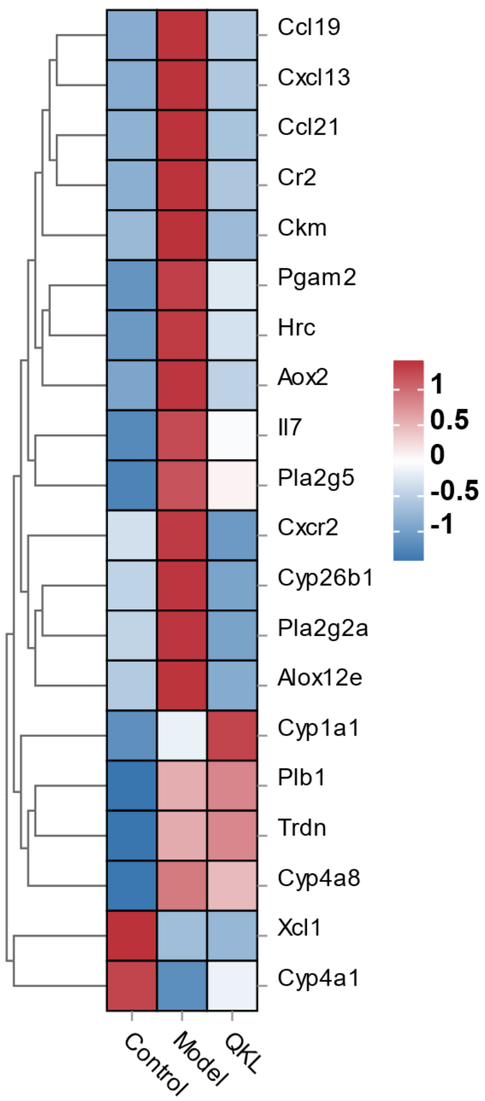

Supplementary Figure 3

Heat map of of relative abundance of 20 core genes.
