# Supplementary material for: Qing-Kai-Ling oral liquid alleviated pneumonia via regulation of intestinal flora and metabolites in rats
Source: Front Microbiol. 2023 Jun 9;14:1194401. doi: 10.3389/fmicb.2023.1194401 (PMC10288885; doi:10.3389/fmicb.2023.1194401)
Supplement: Supplementary file 2 [file Data_Sheet_2.PDF]

Supplementary Table 1. 46 compounds from Qing-Kai-Ling oral liquid by UPLC-Q-TOF-MS in ESI+ and ESI- modes.

Peak No	Retention time (min)	Compound	m/z	Formula	Adducts
1	0.95	L-threonine	119.0582	$C_4H_9NO_3$	M-H
2	0.98	L-arginine	174.1117	$C_6H_{14}N_4O_2$	M+H
3	1.07	Quinic acid	192.0634	$C_7H_{12}O_6$	M-H
4	1.09	Proline	115.0633	$C_5H_9NO_2$	M+H
5	1.17	Sucrose	342.1162	$C_{12}H_{22}O_{11}$	M-H
6	1.22	L-aspartic acid	133.0375	$C_4H_7NO_4$	M+H
7	1.27	Valine	117.0790	$C_5H_{11}NO_2$	M+H
8	1.61	Uridine	244.0695	$C_9H_{12}N_2O_6$	M-H
9	1.65	L-tyrosine	181.0739	$C_9H_{11}NO_3$	M+H
10	5.53	Adenosine	267.0968	$C_{10}H_{13}N_5O_4$	M+H
11	5.54	Adenine	135.0545	$C_5H_5N_5$	M-H
11	5.54	Adenine	135.0545	$C_5H_5N_5$	M+H
12	5.60	Cinnamic acid	148.0524	$C_9H_8O_2$	M-H
13	5.61	Guanosine	283.0917	$C_{10}H_{13}N_5O_5$	M-H
14	6.61	Shanzhiside	392.1319	$C_{16}H_{24}O_{11}$	M-H
15	6.94	Gardoside	374.1213	$C_{16}H_{22}O_{10}$	M-H
16	7.29	L-glutamic acid	147.0532	$C_5H_9NO_4$	M+H
17	7.41	Chlorogenic acid	354.0951	$C_{16}H_{18}O_9$	M-H
18	7.47	Caffeic acid	180.0423	$C_9H_8O_4$	M-H
18	7.47	Caffeic acid	180.0423	$C_9H_8O_4$	M+H
19	7.88	4-Hydroxybenzoic acid	138.0317	$C_7H_6O_3$	M-H
20	7.93	Geniposidic acid	374.1213	$C_{16}H_{22}O_{10}$	M-H
21	8.03	L-tryptophan	204.0899	$C_{11}H_{12}N_2O_2$	M+H
22	8.11	Mussaenosidic acid	376.1370	$C_{16}H_{24}O_{10}$	M-H
23	8.45	Loganic acid	376.1370	$C_{16}H_{24}O_{10}$	M-H

24	8.75	Monotropein	390.1162	$C_{16}H_{22}O_{11}$	M-H
25	9.09	Secologanic acid	374.1213	$C_{16}H_{22}O_{10}$	M-H
26	9.11	Neochlorogenic acid	354.0951	$C_{16}H_{18}O_9$	M-H
27	9.35	Ferulic acid	194.0579	$C_{10}H_{10}O_4$	M-H
27	9.35	Ferulic acid	194.0579	$C_{10}H_{10}O_4$	M+H
28	9.57	Genipin-1-β-D-gentiobioside	550.1898	$C_{23}H_{34}O_{15}$	M+H
29	10.12	Geniposide	388.1370	$C_{17}H_{24}O_{10}$	M-H
30	11.24	Gardenoside	404.1319	$C_{17}H_{24}O_{11}$	M-H
31	11.28	Feruloylquinic acid	368.1107	$C_{17}H_{20}O_9$	M-H
32	11.66	_	_	_	M-H
33	13.24	Rutin	610.1534	$C_{27}H_{30}O_{16}$	M-H
34	13.75	Luteolin	286.0477	$C_{15}H_{10}O_6$	M-H
35	14.21	Cryptochlorogenic acid	354.0951	$C_{16}H_{18}O_9$	M-H
36	14.32	Phenylalanine	165.0790	$C_9H_{11}NO_2$	M+H
37	16.64	Baicalin	446.0849	$C_{21}H_{18}O_{11}$	M-H
38	18.48	Chrysin	254.0579	$C_{15}H_{10}O_4$	M-H
39	18.61	Wogonoside	460.1006	$C_{22}H_{20}O_{11}$	M-H
40	23.02	3,6,7,12-tetrahydroxycholanoic acid	424.2825	$C_{24}H_{40}O_6$	M-H
41	24.29	3,12-dihydroxy-7-ketocholanic acid	406.2719	$C_{24}H_{38}O_5$	M-H
41	24.29	3,12-dihydroxy-7-ketocholanic acid	406.2719	$C_{24}H_{38}O_5$	M+H
42	24.34	3-hydroxy-7,12-diketocholanoic acid	404.2563	$C_{24}H_{36}O_5$	M-H
43	25.68	Hyocholic Acid	408.2876	$C_{24}H_{40}O_5$	M-H
43	25.68	Hyocholic Acid	408.2876	$C_{24}H_{40}O_5$	M+H
44	26.64	Cholic acid	408.2876	$C_{24}H_{40}O_5$	M-H
44	26.64	Cholic acid	408.2876	$C_{24}H_{40}O_5$	M+H
45	27.66	Hyodeoxycholic acid	392.2927	$C_{24}H_{40}O_4$	M-H
45	27.66	Hyodeoxycholic acid	392.2927	$C_{24}H_{40}O_4$	M+H
46	28.55	_	_	_	M-H
47	31.16	Chenodeoxycholic acid	392.2927	$C_{24}H_{40}O_4$	M-H
47	31.16	Chenodeoxycholic acid	392.2927	$C_{24}H_{40}O_4$	M+H

48	31.70	Deoxycholic acid	392.2927	$C_{24}H_{40}O_4$	M-H

Supplementary Table 2 Spearman correlation *P* value of 16 discriminant taxa.

	Mucispirillum	Corynebacterium_1	Jeotgalicoccus	Staphylococcus	Candidatus_Stoquefichus	Paenalcaligenes	Facklamia	Aerococcus
Mucispirillum	--	0.0020	0.0049	0.0014	0.3329	0.0005	0.2006	0.0396
Corynebacterium_1	0.0020	--	0.0022	0.0036	0.3522	0.0020	0.0344	0.0060
Jeotgalicoccus	0.0049	0.0022	--	0.0001	0.0462	0.0037	0.0274	0.0020
Staphylococcus	0.0014	0.0036	0.0001	--	0.0296	0.0009	0.0153	0.0003
Candidatus_Stoquefichus	0.3329	0.3522	0.0462	0.0296	--	0.2906	0.0866	0.0296
Paenalcaligenes	0.0005	0.0020	0.0037	0.0009	0.2906	--	0.0172	0.0040
Facklamia	0.2006	0.0344	0.0274	0.0153	0.0866	0.0172	--	0.0000
Aerococcus	0.0396	0.0060	0.0020	0.0003	0.0296	0.0040	0.0000	--
Ruminiclostridium_1	0.0079	0.0660	0.0113	0.0048	0.0521	0.0017	0.0866	0.0296
Parasutterella	0.0019	0.0057	0.1156	0.0890	0.9131	0.0163	0.3820	0.1882
Bifidobacterium	0.4813	0.7514	0.3104	0.1685	0.0195	0.6927	0.3990	0.1963
Negativibacillus	0.1837	0.0816	0.4671	0.3725	0.7625	0.5314	0.7928	0.4822
Dorea	0.3768	0.0897	0.1739	0.0999	0.2111	0.1023	0.0164	0.0267
Ruminococcus_torques_group	0.1380	0.0528	0.2351	0.1737	0.2841	0.4145	0.4738	0.2096
Desulfovibrio	0.2005	0.3403	0.0392	0.0241	0.0003	0.2334	0.1189	0.0325
Globicatella	0.0282	0.0148	0.0018	0.0012	0.0404	0.0282	0.0184	0.0012

Continuation table

	Ruminiclostridium_1	Parasutterella	Bifidobacterium	Negativibacillus	Dorea	Ruminococcus_torques_group	Desulfovibrio	Globicatella
Mucispirillum	0.0079	0.0019	0.4813	0.1837	0.3768	0.1380	0.2005	0.0282
Corynebacterium_1	0.0660	0.0057	0.7514	0.0816	0.0897	0.0528	0.3403	0.0148
Jeotgalicoccus	0.0113	0.1156	0.3104	0.4671	0.1739	0.2351	0.0392	0.0018
Staphylococcus	0.0048	0.0890	0.1685	0.3725	0.0999	0.1737	0.0241	0.0012
Candidatus_Stoquefichus	0.0521	0.9131	0.0195	0.7625	0.2111	0.2841	0.0003	0.0404
Paenalcaligenes	0.0017	0.0163	0.6927	0.5314	0.1023	0.4145	0.2334	0.0282

Facklamia	0.0866	0.3820	0.3990	0.7928	0.0164	0.4738	0.1189	0.0184
Aerococcus	0.0296	0.1882	0.1963	0.4822	0.0267	0.2096	0.0325	0.0012
Ruminiclostridium_1	--	0.1552	0.4148	0.7625	0.2111	0.5405	0.0693	0.0968
Parasutterella	0.1552	--	0.9186	0.0424	0.7271	0.0815	0.7874	0.1793
Bifidobacterium	0.4148	0.9186	--	0.9074	0.7514	0.1662	0.0051	0.0321
Negativibacillus	0.7625	0.0424	0.9074	--	0.9602	0.0345	0.9866	0.3791
Dorea	0.2111	0.7271	0.7514	0.9602	--	0.3544	0.5748	0.1467
Ruminococcus_torques_group	0.5405	0.0815	0.1662	0.0345	0.3544	--	0.3577	0.1076
Desulfovibrio	0.0693	0.7874	0.0051	0.9866	0.5748	0.3577	--	0.0119
Globicatella	0.0968	0.1793	0.0321	0.3791	0.1467	0.1076	0.0119	--

Supplementary Table 3 Spearman correlation *P* value between 16 differential genera and 7 indicators.

	IL-1β	IL-10	MCP-1	TNF-α	SOD	NO	MPO
Mucispirillum	0.7724	0.0016	0.3631	0.2881	0.0390	0.0006	0.0495
Corynebacterium_1	0.6021	0.0682	0.3423	0.2408	0.0019	0.0130	0.0019
Jeotgalicoccus	0.2756	0.0919	0.0415	0.0338	0.0003	0.0600	0.0022
Staphylococcus	0.1598	0.0124	0.0436	0.0304	0.0026	0.0027	0.0020
Candidatus_Stoquefichus	0.3651	0.0246	0.1849	0.0136	0.3849	0.0807	0.0715
Psychrobacter	0.4705	0.2253	0.2081	0.3618	0.1826	0.0303	0.3306
Paenalcaligenes	0.3631	0.0050	0.1728	0.1961	0.0110	0.0009	0.0711
Facklamia	0.0273	0.4213	0.0236	0.0919	0.0059	0.0663	0.0669
Aerococcus	0.2030	0.3418	0.1229	0.1721	0.0060	0.0770	0.0043
Ruminiclostridium_1	0.6433	0.0031	0.0739	0.2563	0.0176	0.0326	0.1098
Parasutterella	0.9898	0.0127	0.6610	0.6066	0.3513	0.0015	0.2535
Bifidobacterium	0.7621	0.2493	0.3079	0.2924	0.7954	0.0682	0.1245
Dorea	0.2756	0.1659	0.7787	0.1406	0.2756	0.0893	0.3306
Ruminococcus_torques_group	0.7534	0.2176	0.3593	0.3428	0.0999	0.0614	0.0341
Desulfovibrio	0.2511	0.0434	0.0630	0.0149	0.2933	0.0314	0.0809
Globicatella	0.7819	0.1308	0.6018	0.1829	0.1638	0.0124	0.0040

Supplementary Table 4. 64 metabolites between the Control group and the Model group.

No	Compound	Retention time (min)	m/z	Formula	Anova (p)	HMDB ID	Trend (Control /Model)	Class
1	Hippuric acid	3.6913	160.0407	$C_9H_9NO_3$	0.0267	HMDB0000714	down	Benzene and substituted derivatives
2	Schleicherastatin 6	14.1448	475.3418	$C_{28}H_{46}O_3$	0.0005	HMDB0035513	up	Bile acids and derivatives
3	Ursocholic acid	11.9243	389.2690	$C_{24}H_{40}O_5$	0.0014	HMDB0000917	up	Bile acids and derivatives
4	3beta,7alpha-Dihydroxy-5-cholestenoate	13.3479	431.3166	$C_{27}H_{44}O_4$	0.0066	HMDB0012454	up	Bile acids and derivatives
5	27-Deoxy-5b-cyprinol	12.8640	417.3362	$C_{27}H_{48}O_4$	0.0202	HMDB0001231	up	Bile acids and derivatives
6	L-Tyrosine	4.2959	361.1397	$C_9H_{11}NO_3$	0.0015	HMDB0000158	down	Carboxylic acids and derivatives
7	Asparaginyl-Hydroxyproline	8.9927	489.1942	$C_9H_{15}N_3O_5$	0.0271	HMDB0028732	up	Carboxylic acids and derivatives
8	N-a-Acetylcitrulline	14.4435	433.2045	$C_8H_{15}N_3O_4$	0.0491	HMDB0000856	up	Carboxylic acids and derivatives
9	7a-Hydroxy-cholestene-3-one	13.7603	445.3319	$C_{27}H_{44}O_2$	0.0065	HMDB0001993	up	Cholestane steroids
10	Ascorbic acid	0.9231	175.0248	$C_6H_8O_6$	0.0000	HMDB0000044	down	Dihydrofurans
11	11-Hydroxyeicosatetraenoate glyceryl ester	6.5586	393.2642	$C_{23}H_{38}O_5$	0.0016	HMDB0012530	up	Endocannabinoids
12	Prostaglandin G2	6.0747	349.2010	$C_{20}H_{32}O_6$	0.0080	HMDB0003235	up	Fatty Acyls
13	Alpha-linolenyl carnitine	13.8317	402.3005	$C_{25}H_{43}NO_4$	0.0182	HMDB0006319	up	Fatty Acyls
14	L-Palmitoylcarnitine	13.8246	380.3171	$C_{23}H_{45}NO_4$	0.0302	HMDB0000222	up	Fatty Acyls
15	Latanoprost	10.0955	431.2802	$C_{26}H_{40}O_5$	0.0303	HMDB0014792	up	Fatty Acyls
16	11,14,17-Eicosatrienoic acid	13.6538	305.2482	$C_{20}H_{34}O_2$	0.0377	HMDB0060039	up	Fatty Acyls
17	Linoleic acid	13.3764	279.2331	$C_{18}H_{32}O_2$	0.0471	HMDB0000673	up	Fatty Acyls
18	Enterolactone	7.0144	297.1133	$C_{18}H_{18}O_4$	0.0024	HMDB0006101	up	Furanoid lignans
19	MG(0:0/22:5(4Z,7Z,10Z,13Z,16Z)/0:0)	12.4296	403.2845	$C_{25}H_{40}O_4$	0.0295	HMDB0011555	up	Glycerolipids
20	LysoPC(17:0)	12.8140	490.3305	$C_{25}H_{52}NO_7P$	0.0260	HMDB0012108	up	Glycerophospholipids

21	PE(18:1(9Z)/18:3(9Z,12Z,15Z))	13.3621	738.5078	$C_{41}H_{74}NO_8P$	0.0359	HMDB0009062	down	Glycerophospholipids
22	PC(14:1(9Z)/20:1(11Z))	13.3764	802.5619	$C_{42}H_{80}NO_8P$	0.0081	HMDB0007912	down	Glycerophospholipids
23	Beta-Cortolone	10.2448	347.2224	$C_{21}H_{34}O_5$	0.0463	HMDB0013221	up	Hydroxysteroids
24	Paraxanthine	0.8095	161.0463	$C_7H_8N_4O_2$	0.0341	HMDB0001860	down	Imidazopyrimidines
25	L-Tryptophan	5.1642	407.1734	$C_{11}H_{12}N_2O_2$	0.0036	HMDB0000929	down	Indoles and derivatives
26	3-Methyldioxyindole	3.8757	162.0565	$C_9H_9NO_2$	0.0235	HMDB0004186	down	Indoles and derivatives
27	4-Hydroxycyclohexylcarboxylic acid	5.3064	287.1490	$C_7H_{12}O_3$	0.0129	HMDB0001988	down	Organooxygen compounds
28	DHAP(10:0)	5.0642	305.1153	$C_{13}H_{25}O_7P$	0.0207	HMDB0011675	up	Organooxygen compounds
29	D-Glucose	0.9588	161.0454	$C_6H_{12}O_6$	0.0293	HMDB0000122	down	Organooxygen compounds
30	2-Heptanone	12.9563	227.2015	$C_7H_{14}O$	0.0402	HMDB0003671	up	Organooxygen compounds
31	Isovalerylglucuronide	0.9730	277.0903	$C_{11}H_{18}O_8$	0.0372	HMDB0002091	down	Organooxygen compounds
32	13'-Carboxy-alpha-tocopherol	14.1448	459.3477	$C_{29}H_{48}O_4$	0.0002	HMDB0012555	up	Prenol lipids
33	Retinoyl b-glucuronide	5.3564	475.2343	$C_{26}H_{36}O_8$	0.0025	HMDB0003141	down	Prenol lipids
34	Xanthurenic acid	1.1866	204.0302	$C_{10}H_7NO_4$	0.0058	HMDB0000881	down	Quinolines and derivatives
35	16-a-Hydroxypregnenolone	6.6943	377.2321	$C_{21}H_{32}O_3$	0.0052	HMDB0000315	up	Steroids and steroid derivatives
36	D-Urobilin	5.3772	569.2743	$C_{33}H_{40}N_4O_6$	0.0093	HMDB0004161	down	Tetrapyrroles and derivatives
37	Bilirubin	5.3987	583.2580	$C_{33}H_{36}N_4O_6$	0.0168	HMDB0000054	down	Tetrapyrroles and derivatives
38	Mesobilirubinogen	5.9611	591.3174	$C_{33}H_{44}N_4O_6$	0.0168	HMDB0001898	down	Tetrapyrroles and derivatives
39	Sphinganine	10.1106	302.3053	$C_{18}H_{39}NO_2$	0.0292	HMDB0000269	up	Amines
40	Exemestane	4.6026	319.1658	$C_{20}H_{24}O_2$	0.0144	HMDB0015125	down	Androstane steroids
41	Phenyllactic acid	7.3356	149.0599	$C_9H_{10}O_3$	0.0110	HMDB0000779	up	Benzene and substituted derivatives
42	3-Hydroxyanthranilic acid	1.9699	154.0502	$C_7H_7NO_3$	0.0442	HMDB0001476	down	Benzene and substituted derivatives
43	m-Xylene	13.3560	107.0858	C_8H_{10}	0.0018	HMDB0059810	up	Benzene and substituted derivatives
44	Sulfolithocholic acid	4.4605	474.2886	$C_{24}H_{40}O_6S$	0.0233	HMDB0000907	down	Bile acids and derivatives
45	Deoxycholic acid	8.7585	415.2826	$C_{24}H_{40}O_4$	0.0388	HMDB0000626	up	Bile acids and derivatives
46	7-a,27-Dihydroxycholesterol	13.2138	441.3358	$C_{27}H_{46}O_3$	0.0068	HMDB0006281	up	Bile acids, alcohols and derivatives

47	Nopalinic acid	0.8314	245.1141	$C_{10}H_{18}N_2O_6$	0.0056	HMDB0029437	down	Carboxylic acids and derivatives
48	Epi-coprostanol	12.5377	411.3616	$C_{27}H_{48}O$	0.0070	HMDB0001569	up	Cholestane steroids
49	Cholest-5-ene	12.5377	393.3510	$C_{27}H_{46}$	0.0358	HMDB0000941	up	Cholestane steroids
50	Docosahexaenoic acid	12.4376	329.2481	$C_{22}H_{32}O_2$	0.0091	HMDB0002183	up	Fatty Acyls
51	MG(18:2(9Z,12Z)/0:0/0:0)	12.6084	337.2741	$C_{21}H_{38}O_4$	0.0123	HMDB0011568	up	Fatty Acyls
52	MG(0:0/18:3(9Z,12Z,15Z)/0:0)	11.8331	353.2684	$C_{21}H_{36}O_4$	0.0163	HMDB0011540	up	Fatty Acyls
53	Gamma-Linolenic acid	11.6408	279.2326	$C_{18}H_{30}O_2$	0.0187	HMDB0003073	up	Fatty Acyls
54	15(S)-Hydroxyeicosatrienoic acid	12.3169	340.2859	$C_{20}H_{34}O_3$	0.0259	HMDB0005045	up	Fatty Acyls
55	MG(0:0/16:0/0:0)	13.4911	313.2739	$C_{19}H_{38}O_4$	0.0197	HMDB0011533	up	Glycerolipids
56	MG(0:0/18:1(11Z)/0:0)	13.4481	379.2819	$C_{21}H_{40}O_4$	0.0084	HMDB0011536	up	Glycerolipids
57	LysoPC(20:1(11Z))	12.4948	550.3868	$C_{28}H_{56}NO_7P$	0.0363	HMDB0010391	up	Glycerophospholipids
58	Cortexolone	10.2386	347.2215	$C_{21}H_{30}O_4$	0.0497	HMDB0000015	up	Hydroxysteroids
59	Sterol	13.7398	231.2106	$C_{17}H_{28}O$	0.0012	HMDB0060512	up	Hydroxysteroids
60	Indole-3-carboxylic acid	3.8766	162.0553	$C_9H_7NO_2$	0.0200	HMDB0003320	down	Indoles and derivatives
61	4-(2-Aminophenyl)-2,4-dioxobutanoic acid	3.6851	208.0605	$C_{10}H_9NO_4$	0.0071	HMDB0000978	down	Organooxygen compounds
62	All-trans-13,14-dihydroretinol	13.3631	271.2427	$C_{20}H_{32}O$	0.0066	HMDB0011618	up	Prenol lipids
63	Deoxycholic acid 3-glucuronide	5.9405	591.3130	$C_{30}H_{48}O_{10}$	0.0097	HMDB0002596	down	Steroidal glycosides
64	Calcitriol	10.0963	399.3264	$C_{27}H_{44}O_3$	0.0016	HMDB0001903	up	Vitamin D and derivatives

Supplementary Table 5. 52 metabolites between the Model group and the QKL group.

No	Compound	Retention time (min)	m/z	Formula	Anova (p)	HMDB ID	Trend (QKL /Model)	Class
1	3a,7a,12a-Trihydroxy-5b-cholestan-26-al	11.4476	433.3315	$C_{27}H_{46}O_4$	0.0015	HMDB0003533	down	Bile acids and derivatives
2	3b-Hydroxy-5-cholenoic acid	11.6327	373.2748	$C_{24}H_{38}O_3$	0.0060	HMDB0000308	up	Bile acids and derivatives
3	Lithocholic acid	10.6221	375.2902	$C_{24}H_{40}O_3$	0.0070	HMDB0000761	down	Bile acids and derivatives
4	3b,4b,7a,12a-Tetrahydroxy-5b-cholanoic acid	6.8930	469.2798	$C_{24}H_{40}O_6$	0.0119	HMDB0000311	down	Bile acids and derivatives
5	Taurocholic acid	6.6157	496.2733	$C_{26}H_{45}NO_7S$	0.0213	HMDB0000036	up	Bile acids and derivatives
6	20a,22b-Dihydroxycholesterol	13.2479	417.3370	$C_{27}H_{46}O_3$	0.0123	HMDB0006763	down	Bile acids, alcohols and derivatives
7	Hydroxyprolyl-Asparagine	8.5939	489.1939	$C_9H_{15}N_3O_5$	0.0004	HMDB0028858	up	Carboxylic acids and derivatives
8	Valyl-Leucine	3.4633	229.1555	$C_{11}H_{22}N_2O_3$	0.0204	HMDB0029131	up	Carboxylic acids and derivatives
9	Glutaminylglycine	13.8746	405.1731	$C_7H_{13}N_3O_4$	0.0175	HMDB0028797	down	Carboxylic acids and derivatives
10	D-Glutamic acid	0.9659	128.0354	$C_5H_9NO_4$	0.0355	HMDB0003339	up	Carboxylic acids and derivatives
11	Cholesterol	13.5895	431.3515	$C_{27}H_{46}O$	0.0052	HMDB0000067	down	Cholestane steroids
12	2-Arachidonylglycerol	5.7831	423.2742	$C_{23}H_{38}O_4$	0.0264	HMDB0004666	down	Endocannabinoids
13	2-Methylbutyroylcarnitine	6.3949	489.3191	$C_{12}H_{23}NO_4$	0.0007	HMDB0000378	down	Fatty Acyls
14	(9S,10S)-9,10-dihydroxyoctadecanoate	10.8502	297.2435	$C_{18}H_{36}O_4$	0.0038	HMDB0059633	down	Fatty Acyls
15	9,12,13-TriHOME	10.4371	311.2223	$C_{18}H_{34}O_5$	0.0075	HMDB0004708	down	Fatty Acyls
16	9,10-Epoxyoctadecenoic acid	12.2165	295.2275	$C_{18}H_{32}O_3$	0.0114	HMDB0004701	down	Fatty Acyls
17	Pentadecanal	12.8640	225.2223	$C_{15}H_{30}O$	0.0222	HMDB0031078	down	Fatty Acyls
18	(R)-3-Hydroxy-hexadecanoic acid	12.8569	271.2280	$C_{16}H_{32}O_3$	0.0267	HMDB0010734	down	Fatty Acyls
19	MG(0:0/22:6(4Z,7Z,10Z,13Z,16Z,19Z)/0:0)	9.7611	803.5443	$C_{25}H_{38}O_4$	0.0148	HMDB0011557	up	Glycerolipids
20	MG(0:0/20:1(11Z)/0:0)	13.6966	383.3162	$C_{23}H_{44}O_4$	0.0200	HMDB0011543	down	Glycerolipids
21	MG(0:0/24:6(6Z,9Z,12Z,15Z,18Z,21Z)/0:0)	6.6157	489.3205	$C_{28}H_{44}O_4$	0.0280	HMDB0011560	down	Glycerolipids
22	LysoPA(0:0/18:0)	11.4976	483.2733	$C_{21}H_{43}O_7P$	0.0235	HMDB0007850	down	Glycerophospholipids
23	3-hydroxyhexanoic acid	4.4310	131.0715	$C_6H_{12}O_3$	0.0148	HMDB0061652	up	Hydroxy acids and derivatives

24	1H-Indole-3-carboxaldehyde	4.3738	144.0457	C_9H_7NO	0.0082	HMDB0029737	up	Indoles and derivatives
25	Hydroxyphenyllactic acid	3.2211	181.0509	$C_9H_{10}O_4$	0.0485	HMDB0000755	up	Phenylpropanoic acids
26	Pregnenolone	7.6477	361.2375	$C_{21}H_{32}O_2$	0.0006	HMDB0000253	up	Pregnane steroids
27	Retinyl ester	10.2448	347.2224	$C_{20}H_{30}O_2$	0.0450	HMDB0003598	up	Prenol lipids
28	Cortolone-3-glucuronide	12.4439	541.2632	$C_{27}H_{42}O_{11}$	0.0255	HMDB0010320	up	Steroidal glycosides
29	Glycocholic acid	9.1215	483.3423	$C_{26}H_{43}NO_6$	0.0186	HMDB0000138	down	Bile acids and derivatives
30	N1-Acetylspermidine	0.7170	188.1760	$C_9H_{21}N_3O$	0.0044	HMDB0001276	up	Carboximidic acids and derivatives
31	5-L-Glutamyl-taurine	8.2111	255.0652	$C_7H_{14}N_2O_6S$	0.0014	HMDB0004195	up	Carboxylic acids and derivatives
32	Betaine	0.7242	100.0762	$C_5H_{11}NO_2$	0.0026	HMDB0000043	up	Carboxylic acids and derivatives
33	N6,N6,N6-Trimethyl-L-lysine	0.7384	171.1496	$C_9H_{20}N_2O_2$	0.0033	HMDB0001325	up	Carboxylic acids and derivatives
34	Leucyl-phenylalanine	4.5741	279.1700	$C_{15}H_{22}N_2O_3$	0.0413	HMDB0013243	up	Carboxylic acids and derivatives
35	Leucyl-Lysine	0.9594	260.1964	$C_{12}H_{25}N_3O_3$	0.0425	HMDB0028934	up	Carboxylic acids and derivatives
36	Histidinyl-Cysteine	3.9409	259.0850	$C_9H_{14}N_4O_3S$	0.0123	HMDB0028882	down	Carboxylic acids and derivatives
37	7-Dehydrodesmosterol	13.2488	383.3289	$C_{27}H_{42}O$	0.0064	HMDB0003896	down	Cholestane steroids
38	3a,7a-Dihydroxy-5b-cholestane	9.5839	427.3547	$C_{27}H_{48}O_2$	0.0132	HMDB0006893	down	Cholestane steroids
39	7-Dehydrocholesterol	7.8694	407.3300	$C_{27}H_{44}O$	0.0032	HMDB0000032	down	Cholestane steroids
40	PGF2a ethanolamide	6.1614	415.3153	$C_{22}H_{39}NO_5$	0.0039	HMDB0013628	up	Fatty Acyls
41	17-HDoHE	6.4888	327.2316	$C_{22}H_{32}O_3$	0.0220	HMDB0010213	down	Fatty Acyls
42	11Z-Eicosenoic acid	13.8541	328.3209	$C_{20}H_{38}O_2$	0.0245	HMDB0002231	down	Fatty Acyls
43	trans-Hexadec-2-enoyl carnitine	10.2386	415.3516	$C_{23}H_{43}NO_4$	0.0335	HMDB0006317	down	Fatty Acyls
44	12S-HHT	11.3778	263.2006	$C_{17}H_{28}O_3$	0.0458	HMDB0012535	down	Fatty Acyls
45	MG(18:0e/0:0/0:0)	13.3560	367.3166	$C_{21}H_{44}O_3$	0.0260	HMDB0011143	down	Glycerolipids
46	LysoPC(18:1(9Z))	8.7228	504.3438	$C_{26}H_{52}NO_7P$	0.0282	HMDB0002815	up	Glycerophospholipids
47	PC(14:0/16:1(9Z))	13.9964	721.5512	$C_{38}H_{74}NO_8P$	0.0293	HMDB0007870	down	Glycerophospholipids
48	5-Hydroxyindoleacetic acid	4.3747	192.0655	$C_{10}H_9NO_3$	0.0092	HMDB0000763	up	Indoles and derivatives
49	Putrescine	6.7660	89.1077	$C_4H_{12}N_2$	0.0021	HMDB0001414	up	Organonitrogen compounds
50	4,4-Dimethyl-5a-cholesta-8-en-3b-ol	13.4553	397.3811	$C_{29}H_{50}O$	0.0061	HMDB0006840	down	Prenol lipids
51	Cholesteryl acetate	12.2175	429.3717	$C_{29}H_{48}O_2$	0.0048	HMDB0003822	down	Steroids and steroid derivatives
52	24-Hydroxycalcitriol	8.4311	455.3150	$C_{27}H_{44}O_4$	0.0016	HMDB0006228	down	Vitamin D and derivatives

Supplementary Table 6. 24 metabolites among the Control, Model and QKL groups.

No	Metabolites	HMDB	class
1	Sebacic acid	HMDB0000792	Fatty Acyls
2	Cholic acid	HMDB0000619	Steroids and steroid derivatives
3	12-Ketodeoxycholic acid	HMDB0000328	Steroids and steroid derivatives
4	7-Sulfocholic acid	HMDB0002421	Steroids and steroid derivatives
5	3-Sulfodeoxycholic acid	HMDB0002504	-
6	7-Ketodeoxycholic acid	HMDB0000391	Steroids and steroid derivatives
7	Deoxycholic acid glycine conjugate	HMDB0000631	Steroids and steroid derivatives
8	Nutriacholic acid	HMDB0000467	Steroids and steroid derivatives
9	8,11,14-Eicosatrienoic acid	HMDB0002925	Fatty Acyls
10	Adrenic acid	HMDB0002226	Fatty Acyls
11	Arachidonic acid	HMDB0001043	Fatty Acyls
12	Coprocholic acid	HMDB0000601	Steroids and steroid derivatives
13	Nonadecanoic acid	HMDB0000772	Fatty Acyls
14	7a,12a-Dihydroxy-3-oxo-4-cholenoic acid	HMDB0000447	Steroids and steroid derivatives
15	alpha-CEHC	HMDB0001518	Benzopyrans
16	Cortol	HMDB0003180	Steroids and steroid derivatives
17	LysoPC(20:0/0:0)	HMDB0010390	Glycerophospholipids
18	LysoPA(18:0e/0:0)	HMDB0011144	Glycerophospholipids
19	MG(0:0/22:2(13Z,16Z)/0:0)	HMDB0011553	Glycerolipids
20	Cervonoyl ethanolamide	HMDB0013627	Fatty Acyls
21	Sulfolithocholylglycine	HMDB0002639	Steroids and steroid derivatives
22	Tetrahydrocorticosterone	HMDB0000268	Steroids and steroid derivatives
23	Heptadecanoyl carnitine	HMDB0006210	Fatty Acyls
24	Dihomo-gamma-Linolenoyl ethanolamide	HMDB0013625	Amines

Supplementary Table 7 Spearman correlation *P* value among the microbiome, biochemical indices and metabolome.

	Mucispirillum	Corynebacterium_1	Jeotgalicoccus	Staphylococcus	Candidatus_Stoquefichus	Psychrobacter	Paenalcaligenes	Facklamia
Sebacic acid	0.0076	0.0202	0.0011	0.0000	0.0373	0.0800	0.0017	0.0044
Cholic acid	0.0051	0.0003	0.0139	0.0064	0.1984	0.0220	0.0067	0.0413
12-Ketodeoxycholic acid	0.0051	0.0003	0.0139	0.0064	0.1984	0.0220	0.0067	0.0413
7-Ketodeoxycholic acid	0.0009	0.0126	0.0016	0.0004	0.0626	0.0479	0.0020	0.0774
Deoxycholic acid glycine conjugate	0.0153	0.0114	0.0153	0.0129	0.0525	0.0332	0.0390	0.1513
Nutriacholic acid	0.0051	0.0008	0.0220	0.0064	0.1598	0.0126	0.0067	0.0413
7a,12a-Dihydroxy-3-oxo-4-cholenoic acid	0.0044	0.0625	0.0261	0.0032	0.0525	0.0307	0.0110	0.1347
Cervonoyl ethanolamide	0.0086	0.0019	0.0240	0.0057	0.1443	0.0220	0.0051	0.0202
Tetrahydrocorticosterone	0.0208	0.0283	0.0139	0.0129	0.1007	0.1182	0.0814	0.3513
Heptadecanoyl carnitine	0.0059	0.0240	0.0126	0.0005	0.0304	0.0666	0.0051	0.0469
Dihomo-gamma-Linolenoyl ethanolamide	0.0359	0.1908	0.1517	0.0093	0.0129	0.0899	0.0188	0.0509
7-Sulfocholic acid	0.1558	0.2551	0.3306	0.0453	0.0144	0.0261	0.0986	0.0287
3-Sulfodeoxycholic acid	0.1558	0.1517	0.1745	0.0330	0.0015	0.1745	0.1048	0.0669
8,11,14-Eicosatrienoic acid	0.1399	0.3911	0.4038	0.1007	0.0267	0.0548	0.3236	0.4183
Arachidonic acid	0.4643	0.9312	0.7954	0.1894	0.0506	0.2551	0.5274	0.3372
Coprocholic acid	0.0423	0.0952	0.1993	0.0129	0.0144	0.0074	0.0575	0.0552
alpha-CEHC	0.0986	0.2969	0.5273	0.0978	0.0647	0.0026	0.2005	0.2249
LysoPA(18:0e/0:0)	0.9425	0.8970	0.5717	0.9083	0.5068	0.0849	0.8662	0.3442
Sulfolithocholylglycine	0.2413	0.3423	0.4568	0.0626	0.0470	0.2453	0.1180	0.0395
Adrenic acid	0.0869	0.1745	0.1667	0.0093	0.0213	0.1063	0.0359	0.0108
Nonadecanoic acid	0.5603	0.7954	0.8970	0.5614	0.2372	0.1591	0.9616	0.9284
LysoPC(20:0/0:0)	0.0664	0.0307	0.0283	0.0014	0.0584	0.2861	0.0208	0.0040
MG(0:0/22:2(13Z,16Z)/0:0)	0.0044	0.0220	0.0168	0.0010	0.0373	0.0202	0.0153	0.0860
Cortol	0.0014	0.0082	0.0185	0.0001	0.0488	0.2170	0.0006	0.0450

Continuation table

	Aerococcus	Ruminiclostridium_1	Parasutterella	Bifidobacterium	Dorea	Ruminococcus_torques_group	Desulfovibrio	Globicatella
Sebacic acid	0.0151	0.0129	0.1347	0.1063	0.1245	0.1144	0.0035	0.0330
Cholic acid	0.0544	0.1443	0.0020	0.3191	0.0479	0.0106	0.2267	0.0203
12-Ketodeoxycholic acid	0.0544	0.1443	0.0020	0.3191	0.0479	0.0106	0.2267	0.0203
7-Ketodeoxycholic acid	0.1067	0.0093	0.0089	0.1063	0.3079	0.0581	0.0150	0.0470
Deoxycholic acid glycine conjugate	0.1519	0.2125	0.0059	0.0952	0.1517	0.0247	0.0400	0.0104
Nutriacholic acid	0.0358	0.1067	0.0020	0.3423	0.1310	0.0291	0.1437	0.0255
7a,12a-Dihydroxy-3-oxo-4-cholenoic acid	0.2635	0.0604	0.0059	0.0625	0.2170	0.0508	0.0129	0.0839
Cervonoyl ethanolamide	0.0233	0.0949	0.0044	0.3079	0.1310	0.0660	0.1108	0.0233
Tetrahydrocorticosterone	0.1894	0.2271	0.0362	0.2081	0.6021	0.0919	0.0480	0.0436
Heptadecanoyl carnitine	0.0839	0.0093	0.0273	0.1377	0.1517	0.0441	0.0248	0.1162
Dihomo-gamma-Linolenoyl ethanolamide	0.1806	0.0788	0.1347	0.0479	0.0082	0.1144	0.0059	0.0788
7-Sulfocholic acid	0.1763	0.1519	0.0952	0.0051	0.0220	0.0208	0.0173	0.1037
3-Sulfodeoxycholic acid	0.1558	0.0866	0.1347	0.0153	0.0126	0.0341	0.0238	0.0626
8,11,14-Eicosatrienoic acid	0.4121	0.3916	0.1193	0.0000	0.3079	0.0166	0.0059	0.0291
Arachidonic acid	0.6772	0.6183	0.3372	0.0004	0.1908	0.0867	0.0344	0.2635
Coprocholic acid	0.1598	0.1519	0.0330	0.0103	0.0153	0.0013	0.0190	0.0669
alpha-CEHC	0.4474	0.3586	0.0330	0.0040	0.1063	0.0018	0.0385	0.1405
LysoPA(18:0e/0:0)	0.7642	0.7730	0.2361	0.1591	0.2861	0.2028	0.5571	0.5694
Sulfolithocholylglycine	0.2528	0.3984	0.1646	0.0220	0.0022	0.0972	0.0859	0.1195
Adrenic acid	0.0584	0.0978	0.2305	0.0065	0.0168	0.0660	0.0080	0.0358
Nonadecanoic acid	1.0000	0.9175	0.1933	0.0040	0.6806	0.0441	0.1822	0.2173
LysoPC(20:0/0:0)	0.0255	0.1806	0.1513	0.0479	0.0074	0.0508	0.0671	0.0185
MG(0:0/22:2(13Z,16Z)/0:0)	0.0739	0.0893	0.0489	0.0709	0.1377	0.0217	0.0063	0.0213
Cortol	0.0403	0.0017	0.0469	0.2170	0.0513	0.0600	0.0761	0.0814

Continuation table

	IL-1β	IL-10	MCP-1	TNF-a	SOD	NO	MPO
Sebacic acid	0.0240	0.0396	0.0168	0.0079	0.0139	0.0034	0.0415
Cholic acid	0.4568	0.0682	0.3663	0.1338	0.0332	0.0044	0.0126
12-Ketodeoxycholic acid	0.4568	0.0682	0.3663	0.1338	0.0332	0.0044	0.0126

7-Ketodeoxycholic acid	0.2969	0.0060	0.0114	0.0439	0.0415	0.0050	0.0220
Deoxycholic acid glycine conjugate	0.4299	0.0411	0.1826	0.0338	0.1517	0.0090	0.0153
Nutriacholic acid	0.2170	0.0144	0.2262	0.0723	0.0800	0.0006	0.0153
7a,12a-Dihydroxy-3-oxo-4-cholenoic acid	0.1310	0.0064	0.0240	0.0234	0.2170	0.0020	0.0709
Cervonoyl ethanolamide	0.1245	0.0144	0.1517	0.0598	0.1063	0.0002	0.0261
Tetrahydrocorticosterone	0.2262	0.0112	0.0952	0.0109	0.2262	0.0303	0.0082
Heptadecanoyl carnitine	0.0899	0.0008	0.0082	0.0134	0.0666	0.0064	0.0114
Dihomo-gamma-Linolenoyl ethanolamide	0.0479	0.0525	0.3306	0.0325	0.2652	0.0018	0.2861
7-Sulfocholic acid	0.2081	0.1403	0.1245	0.1783	0.4038	0.0057	0.1667
3-Sulfodeoxycholic acid	0.4168	0.0411	0.1310	0.0746	0.2861	0.0426	0.0283
8,11,14-Eicosatrienoic acid	0.8970	0.2395	0.4299	0.4805	0.7787	0.0137	0.1182
Arachidonic acid	0.2756	0.3126	0.1826	0.3146	0.7954	0.0682	0.3079
Coprocholic acid	0.1745	0.0525	0.2262	0.0866	0.2551	0.0013	0.0479
alpha-CEHC	0.4845	0.1858	0.3911	0.3995	0.6175	0.0041	0.1993
LysoPA(18:0e/0:0)	0.5567	0.8797	0.7456	0.9740	0.5567	0.1335	0.9312
Sulfolithocholylglycine	0.0952	0.2253	0.3423	0.1514	0.6488	0.0137	0.2551
Adrenic acid	0.1310	0.1985	0.2453	0.1783	0.2170	0.0023	0.1591
Nonadecanoic acid	0.6175	0.6962	0.5717	0.9566	0.7129	0.2253	0.2756
LysoPC(20:0/0:0)	0.0849	0.1777	0.1446	0.0541	0.0800	0.0130	0.0153
MG(0:0/22:2(13Z,16Z)/0:0)	0.0952	0.0268	0.2170	0.0171	0.0800	0.0010	0.0283
Cortol	0.2356	0.0006	0.1377	0.0892	0.0332	0.0047	0.0074
